# Hydrogen bonding asymmetric star-shape derivative of bile acid leads to supramolecular fibrillar aggregates that wrap into micrometer spheres†
†Electronic supplementary information (ESI) available: Materials and methods, experimental section, and characterization. See DOI: 10.1039/c6sm01329e



**DOI:** 10.1039/c6sm01329e

**Published:** 2016-07-28

**Authors:** Teemu T. T. Myllymäki, Nonappa, Hongjun Yang, Ville Liljeström, Mauri A. Kostiainen, Jani-Markus Malho, X. X. Zhu, Olli Ikkala

**Affiliations:** a Department of Applied Physics , Aalto University , P.O. Box 15100 , FI-00076 AALTO , Finland . Email: nonappa@aalto.fi ; Email: olli.ikkala@aalto.fi; b Department of Chemistry , Université de Montréal , C.P. 6128 , Succursale Centre-ville , Montréal , QC H3C 3J7 , Canada; c Department of Biotechnology and Chemical Technology , Aalto University , P.O. Box 16100 , FI-00076 AALTO , Finland

## Abstract

We report that star-shaped molecules with cholic acid cores asymmetrically grafted by low molecular weight polymers with hydrogen bonding end-groups undergo aggregation to nanofibers, which subsequently wrap into micrometer spherical aggregates with low density cores. Therein the facially amphiphilic cholic acid (CA) is functionalized by four flexible allyl glycidyl ether (AGE) side chains, which are terminated with hydrogen bonding 2-ureido-4[1*H*]pyrimidinone (UPy) end-groups as connected by hexyl spacers, denoted as CA(AGE_6_-C_6_H_12_-UPy)_4_. This wedge-shaped molecule is expected to allow the formation of a rich variety of solvent-dependent structures due to the complex interplay of interactions, enabled by its polar/nonpolar surface-active structure, the hydrophobicity of the CA in aqueous medium, and the possibility to control hydrogen bonding between UPy molecules by solvent selection. In DMSO, the surfactant-like CA(AGE_6_-C_6_H_12_-UPy)_4_ self-assembles into nanometer scale micelles, as expected due to its nonpolar CA apexes, solubilized AGE_6_-C_6_H_12_-UPy chains, and suppressed mutual hydrogen bonds between the UPys. Dialysis in water leads to nanofibers with lateral dimensions of 20–50 nm. This is explained by promoted aggregation as the hydrogen bonds between UPy molecules start to become activated, the reduced solvent dispersibility of the AGE-chains, and the hydrophobicity of CA. Finally, in pure water the nanofibers wrap into micrometer spheres having low density cores. In this case, strong complementary hydrogen bonds between UPy molecules of different molecules can form, thus promoting lateral interactions between the nanofibers, as allowed by the hydrophobic hexyl spacers. The wrapping is illustrated by transmission electron microscopy tomographic 3D reconstructions. More generally, we foresee hierarchically structured matter bridging the length scales from molecular to micrometer scale by sequentially triggering supramolecular interactions.

## Abstract 2

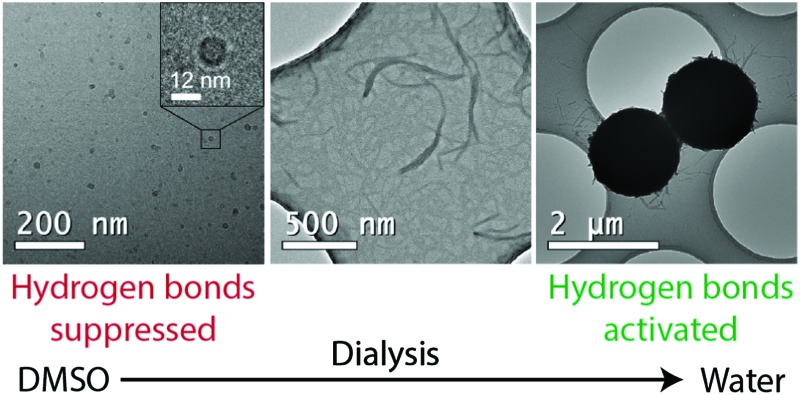
Star-shaped asymmetrically grafted low molecular weight polymers with hydrogen bonding end-groups undergo aggregation to nanofibers, which subsequently wrap into micrometer spherical aggregates.

## Introduction

Bile acids are surface active steroids that have several important biological functions, but they have also attracted extensive interest in materials science and supramolecular chemistry as starting materials for self-assemblies, aggregates, and functionalities.^1–6^ This is driven by their particular chemical structure consisting of four fused alicyclic rings, thus forming a rigid core, which asymmetrically incorporates varying number of hydroxyl groups, as well as a carboxylic acid side chain.^7^ Such a chemical structure leads to facial amphiphilicity, where one side is hydrophilic, whereas the opposing side is hydrophobic. This particular structure leads to a subtle surface-active behaviour in comparison to classic surfactants.^6^

In biological systems, bile acids undergo self-assembly in different kinds of micelles to solubilize hydrophobic compounds, as driven by their amphiphilicity. Therein primary and secondary micellar structures have been described.^3,8^ The propensity for side-by-side packing of bile acids has been recently directly visualized down to the molecular level using atomic force microscopy techniques.^9^ On a larger scale, upon tuning the assembly conditions as well as by chemical and supramolecular functionalization, a wide range of different self-assembled structures can be observed, such as globular aggregates,^10–12^ lamellar structures upon decorating with bulky adamantyl groups,^13^ as well as rod-like, tubular, ribbon-like, and fibrillar structures, also facilitating gelation.^14–32^ The chirality of bile acid allows helicity in self-assembled fibers and ribbons.^33–35^ The solvent quality and incorporation of thermoresponsive functionalizations can trigger different forms of self-assemblies.^19,27,36,37^ Finally, vesicles can also be obtained.^38^ Even more complex bile-acid constructs have been demonstrated by combining *e.g.* peptide segments, allowing hydrogen bonds towards more advanced self-assemblies and aggregates.^39–43^ Relevant to the present context, the asymmetrically positioned hydroxyl groups in bile acids allow construction of low molecular weight polymeric nonionic asymmetric star-shape surfactants upon polymerization of *e.g.* polyethylene glycol where the hydrophobic face of the bile acid forms the apex.^44–47^ Such surfactants can adopt a wedge-like shape, and they can become stimuli responsive due to competing interactions, *i.e.* the interplay of the hydrophobic and solvophilic interactions.

Here we address star-shaped cholic acid (CA) derivatives which form a wedge-shaped architecture due to asymmetrically grafted side chains, which are additionally functionalized using hydrogen bonding end-groups. We expected this architecture to be interesting *e.g.* in surface modifications where the hydrophobic CA-apex could bind on hydrophobic surfaces and the hydrogen bonding moieties at the opposing end could provide supramolecular binding sites. Due to the complex combination of interactions, the behavior turned unexpectedly interesting. In more detail, we use CA, which has been asymmetrically decorated with oligo-allyl glycidyl ether (AGE) chains to allow a wedge-shaped architecture by reacting with a low molecular weight polymer, which has been telechelically modified with the hydrogen bonding motif, 2-ureido-4[1*H*]pyrimidinone (UPy), and separated by hexyl spacers. The asymmetric star-shape polymers are denoted as CA(AGE_6_-C_6_H_12_-UPy)_4_. UPy is feasible in supramolecular constructions, as it allows four complementary hydrogen bonds in end-to-end arrangement, thus facilitating supramolecular assembly, self-healing, and promoted toughness in materials.^48–52^ The dimerization constant exceeds 10^6^ M^–1^ in CHCl_3_,^53,54^ which does not suppress the mutual UPy hydrogen bonds. On the other hand, in highly polar solvents such as dimethyl sulfoxide (DMSO), the dimerization between UPy molecules is suppressed.^55^ Thus the architecture and the strength of the hydrogen bonds between UPy molecules can be tuned upon selecting the solvents. Importantly, two UPy molecules can also form mutual hydrogen bonds in aqueous media, given that they are connected within a local hydrophobic environment,^51,56–58^ as provided by the hexyl spacer in the present case. The fact that novel forms of aggregations and self-assemblies of CA-derivatives could be achieved using asymmetrically side-chain modified cholic acids has recently been indicated by bilosomes with well-defined structures,^43^ where the side chain ends have been modified using crosslinkable sites.

In this work, we investigate the structure formation of CA(AGE_6_-C_6_H_12_-UPy)_4_ first in DMSO and in subsequent dialysis against water. The structures are characterized by transmission electron microscopy (TEM), and scanning electron microscopy (SEM), and supported by dynamic light scattering (DLS) and small-angle X-ray scattering (SAXS).

## Results and discussion

**Fig. 1:**
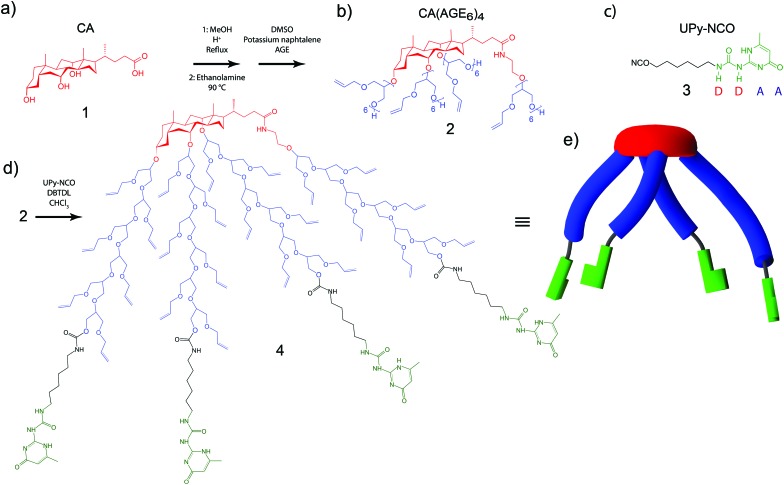
(a) Cholic acid (CA) starting material **1**, (b) CA grafted by four oligo(allyl glycidyl ether) (AGE) chains **2** and (c) 2-ureido-4[1*H*]pyrimidinone isocyanate with hexyl linker (UPy-NCO) **3**. (d) The asymmetric star-shape end-functionalized bile acid derivative CA(AGE_6_-C_6_H_12_-UPy)_4_ **4** and (e) its schematic representation, illustrating that the characteristic four-hydrogen bond supramolecular interactions between UPy molecules cannot be formed between those of the same CA(AGE_6_-C_6_H_12_-UPy)_4_ molecules.

First, CA **1** (Fig. 1a) was asymmetrically grafted with four oligo(allyl glycidyl ether) (AGE) chains *via* anionic polymerization, leading to CA(AGE_6_)_4_ **2** (Fig. 1b), as reported previously (see ESI†).^44^ UPy-isocyanate (UPy-NCO) **3** (Fig. 1c) was synthesized by the reaction of 2-amino-4-hydroxy-6-methylpyrimidinone with a large excess of hexamethylene diisocyanate (see ESI†). CA(AGE_6_)_4_ was then reacted with excess UPy-NCO in the presence of dibutyltin dilaurate (DBTDL) in CHCl_3_, and was purified by precipitating in hexane, centrifugation, and dialysis in CHCl_3_. The final product was denoted as CA(AGE_6_-C_6_H_12_-UPy)_4_ **4**, where Fig. 1d illustrates the characteristic wedge-shaped architecture. It should be noted that the UPy end-groups within a single CA(AGE_6_-C_6_H_12_-UPy)_4_ molecule can form lateral hydrogen bonds, but the characteristic quadruple hydrogen bond end-to-end arrangement is sterically hindered in this case and can take place only between UPy molecules belonging to different CA(AGE_6_-C_6_H_12_-UPy)_4_ molecules. In DMSO, the quadruple hydrogen bonding dimerization between UPy molecules is inhibited by tautomeric changes^54,55^ and the mutual hydrogen bonds are expected to be suppressed therein. The structure (Fig. 1d) also illustrates the ample possibilities for further chemical modification using the available double bonds of AGE. Fig. 1e still illustrates the molecular architecture schematically: the rigid cholic acid core is lipophilic (low polarity) promoting micellar aggregates in polar solvents, the four short asymmetrically grafted AGE_6_ chains are flexible and more polar, and the four hydrogen bonding architectures of UPy are schematically illustrated.

**Fig. 2:**
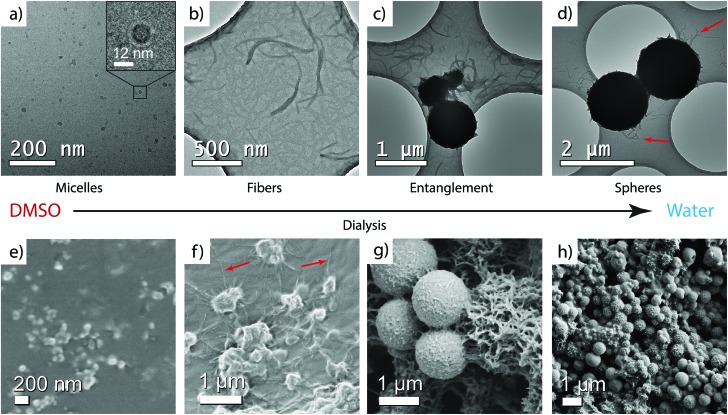
TEM micrographs of CA(AGE_6_-C_6_H_12_-UPy)_4_ **4** upon dialyzing from DMSO to water. (a) Cryo-TEM in DMSO. Dry TEM samples from (b and c) DMSO/water 50/50 v/v, and (d) water (red arrows shows the fibers). SEM micrographs in (e) DMSO, (f) DMSO/water 30/70 v/v, and (g and h) water.

CA(AGE_6_-C_6_H_12_-UPy)_4_ was first dissolved in DMSO (1 mg mL^–1^). Thereafter, the sample was slowly and stepwise dialyzed against water to afford well-defined DMSO/water mixtures (regenerated cellulose membrane, molecular weight cut-off 2000 Da, see ESI† for details). After each addition of water, the system was stirred for 1 h to allow solvent exchange inside the dialysis tube. Eventually, the solvent was completely exchanged to water. Samples for TEM, DLS, and SAXS were taken from DMSO/water mixtures during the dialysis process. The samples in DMSO were vitrified and imaged using cryo-TEM. The water-containing samples were analyzed using conventional TEM of dried samples (Fig. 2, see also the ESI†).

**Fig. 3:**
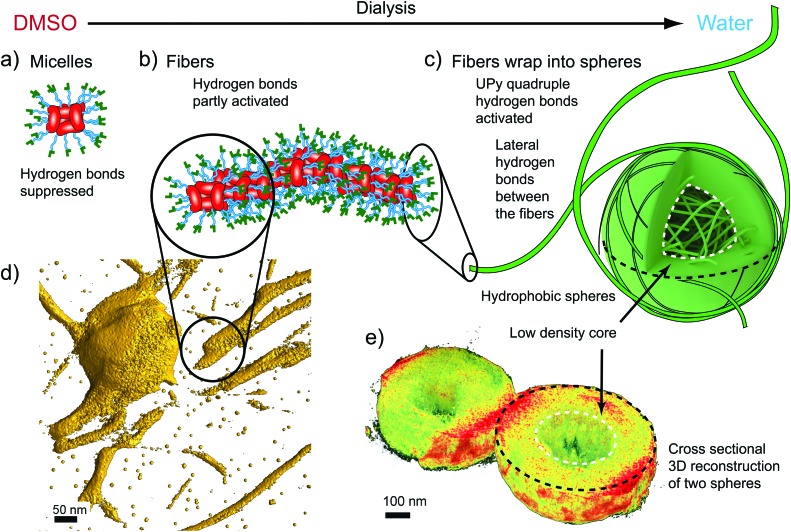
Schematics and electron tomogram snapshots for the hierarchical aggregation of CA(AGE_6_-C_6_H_12_-UPy)_4_. (a) Schematics for the suggested micelles in DMSO, with CA moieties facing the center. (b) Schematics for the nanofibers in DMSO/water mixtures. (c) Fibers wrapped as spheres with low density cores. (d) Electron Tomography reconstruction (3D isosurface) of the fibers (see also Videos S1 and S2, ESI†). (e) Tomographic reconstruction showing the cross-section of two spheres with low density cores.

In DMSO, cryo-TEM qualitatively shows that CA(AGE_6_-C_6_H_12_-UPy)_4_ forms approximately spherical aggregates with the diameter in the range of 10–20 nm (Fig. 2a). SEM shows small aggregates that are larger than those observed in TEM, probably due to the different sample preparation method, involving evaporation of solvent and therefore possible micelle fusion. More quantitative determination is provided by DLS, which indicates the diameter of 11.55 nm for micelles in DMSO (see Fig. S14 and Table S1, ESI†). Also, SAXS of CA(AGE_6_-C_6_H_12_-UPy)_4_ shows a broad correlation peak at the scattering vector magnitude *q* ∼ 0.035–0.045 Å^–1^ corresponding to structural features of 14–18 nm, and is interpreted as the form factor of micelles of CA(AGE_6_-C_6_H_12_-UPy)_4_ (Fig. S15, ESI†). In fact, micelles in DMSO could be expected, due to the surfactant-like wedge shape of CA(AGE_6_-C_6_H_12_-UPy)_4_, where the nonpolar CA apex is on one side of the molecule, forming the micellar core and the DMSO-soluble more polar CA(AGE_6_-C_6_H_12_-UPy)_4_ chains are on the other end, forming the micellar shell. Furthermore, the mutual hydrogen bonds of UPy are expected to be largely suppressed in DMSO, which is expected to promote the observed spherical aggregate shape. The schematic representation of the micelles is shown in Fig. 3a.

Upon addition of water, fibrillar aggregates are observed, as indicated by TEM images of dried samples. Fig. 2b shows nanofibers with lateral dimensions of 20–50 nm. Importantly, the fibrillar aggregates seem to be metastable as they undergo further aggregation to spheres with time (Fig. 2c). The fibers, as well as the initial growth of the wrapped spheres, can be observed also using SEM as shown in Fig. 2f. The fibers were observed using DLS (Fig. S14, ESI†), but the single angle DLS measurements using Contin-analysis do not allow quantitative analysis of particles other than spherical shapes. In SAXS, the form factor corresponding to small micelles starts to gradually disappear as the water content is increased, which agrees with the formation of larger aggregates with high polydispersity. To visualize the fibers better, 3D reconstruction was made from a tilt-series of TEM images (Fig. 3d, see also Video S1, ESI†), according to which a suggestion of fibrillar micelles is presented in Fig. 3b.

Wrapping into spheres is promoted as the water content is further increased in the dialysis, leading to the gradual disappearance of separate fibers. The wrapped spheres become dominant when the solvent is completely exchanged to water. TEM qualitatively suggests that the diameter of the spheres is in the micrometer range (Fig. 1d and Fig. S20, ESI†) and many of them are physically connected by residual nanofibers. The spheres were observed also using SEM, where they formed a space-filling porous network upon drying (Fig. 2g, h and Fig. S25–S28, ESI†). DLS shows that in pure water the mean aggregate size is 888 nm (Fig. S14 and Table S1, ESI†). The 3D reconstruction of spheres shows a dense shell covering a low density core of the spheres (Fig. 3e, see also Fig. S22 and Video S2, ESI†).

Control experiments were performed to gain understanding on the role of the hydrogen bonding UPy motif and the hydrophobic hexyl spacer group in the self-assembly. First CA(AGE_6_)_4_ (Fig. 1b), *i.e.* the material without UPy and the hexyl spacer, was studied in a similar way to CA(AGE_6_-C_6_H_12_-UPy)_4_. Cryo-TEM of CA(AGE_6_)_4_ in DMSO (Fig. S19, ESI†) shows small spherical micellar aggregates with average sizes of 11.6 ± 2.2 nm (Table S2, ESI†). DLS indicates a mean particle diameter of 9.2 nm. These findings are closely reminiscent to those observed for CA(AGE_6_-C_6_H_12_-UPy)_4_ in DMSO. The formation of similar micelles with low polar CA cores with solubilizing side-chain brushes with or without UPy is indirect evidence of inactivated UPy in DMSO. Upon increasing the amount of water in dialysis until complete exchange to water, no fibrils or wrapping into micrometer spherical aggregates are observed. Instead, in pure water small micellar aggregates are observed in TEM (Fig. S17d, ESI†) with a micellar diameter of 35 nm based on DLS (Table S1, ESI†). Another control experiment deals with molecules which do not contain the terminal UPy molecules, *i.e.* where the side chains are terminated only by the hydrophobic hexyl groups, CA(AGE_6_-C_6_H_13_)_4_ (**7**, Fig. S11, ESI†). Upon dialysis from DMSO to water, polydisperse spherical aggregates with diameters roughly from 15 nm to 40 nm were observed using TEM (Fig. S21, ESI†) without any higher level aggregates. The above control experiments suggest that UPy plays a significant role in the formation of fibers and the micrometer scale spheres upon dialysis of CA(AGE_6_-C_6_H_12_-UPy)_4_ from DMSO to water.

The systematic observations in different DMSO/water mixtures for CA(AGE_6_-C_6_H_12_-UPy)_4_, CA(AGE_6_-C_6_H_13_)_4_, and CA(AGE_6_)_4_ allow us to make suggestions for the aggregation behaviors, as schematically shown in Fig. 3. First, the micellar self-assembly of CA(AGE_6_-C_6_H_12_-UPy)_4_ in DMSO arises due to the surfactant-like nature with nonpolar CA cores with the AGE_6_-C_6_H_12_-UPy solubilizing chains (Fig. 3a). The size of CA(AGE_6_-C_6_H_12_-UPy)_4_ micelles in DMSO suggests that there are *ca.* 4–8 molecules involved. Upon adding water, the micelles start to aggregate into fibers. The solvent exchange from DMSO to water involves several effects: the AGE_6_-C_6_H_12_-UPy chains can form progressively increased mutual lateral hydrogen bonds between the neighboring side-chains within the same CA(AGE_6_-C_6_H_12_-UPy)_4_ molecule, and therefore the molecule becomes less conical in shape as the neighboring AGE_6_-C_6_H_12_-UPy chains can become more tightly packed. This can reduce the curvature induced by the surfactant. Upon adding water, the hydrophobicity of CA can also start playing an increasing role. In addition, progressively increased hydrogen bonding between different micelles becomes allowed as facilitated by the UPy groups (Fig. 3b). The outer rim of the nanofibers contains a dense set of UPy groups that can become activated to form strong quadruple hydrogen bonds, thus facilitating lateral adsorption of the fibers to wrap the spheres (Fig. 3c). The wrapped microspheres show thick shells with less dense cores, according to TEM tomography (Fig. 3e, Video S2, ESI†).

Finally, we emphasize the role of the hexyl spacer. Water inhibits UPy dimerization,^54^ but hydrophobicity in the near proximity of the UPy groups can create hydrophobic pockets to allow the formation of UPy quadruple hydrogen bonded dimers.^51,56–58^ We used six carbon long aliphatic linkers to attach UPy to AGE oligomers. Therefore, the CA(AGE_6_-C_6_H_12_-UPy)_4_ molecule and the fibers are insoluble in water, and UPy dimerization is expected between the nanofibers in water. Consequently, the wrapping into spheres in water is expected, as spheres have the smallest surface area to volume ratio. To explain the less dense cores in the spheres, the fibers are not flexible enough to wrap effectively to fully fill the core of the spheres, resulting in a smaller density in the cores as illustrated in Fig. 3c. There are a few related observations in the previous literature. Star-like block-copolymers consisting of PS and PEO-chains asymmetrically connected to an aromatic core lead to cylindrical micelles that can form micrometer spheres having a cylindrical micellar substructure.^59^ This structure was denoted as “cottonballs”. Another approach is based on poly(2-isopropyl-2-oxazoline), which leads to micrometer aggregates due to the thermal trigger by a thermosensitive polymer.^60^ Polymerization-induced block copolymer self-assembly can lead to cylindrical micelles that further form partly fused vesicular structures.^61^

## Conclusions

We have constructed asymmetric star-shaped bile acid derivatives decorated with hydrogen bonding UPy groups at the end of the side-chain brushes. The apex of the wedge-shaped molecule is formed by the nonpolar face of cholic acid, whereas the asymmetric side chains as well as UPy are polar. This makes the molecule behave as a surfactant in the highly polar solvent DMSO to allow micelles. Still, complexity beyond classic surfactants is observed, as depending on the solvent quality by adding water, the hydrogen bonds can be activated, thus leading to solvent dependent aggregation first to nanofibers and then wrapping into microscale spheres. In this way, aggregates can be constructed from the nanoscale to the microscale. We foresee that concepts could be developed in more general direction for solvent triggered hierarchical self-assemblies from the nanoscale upwards, for primary, secondary, tertiary and higher order structures by sequentially activating supramolecular interactions. Achieving such a control of molecular self-assembly up to macroscopic objects might lead to fascinating nanostructured and responsive materials.
