# Supplementary material for: Hydrogen bonding asymmetric star-shape derivative of bile acid leads to supramolecular fibrillar aggregates that wrap into micrometer spheres
Source: Soft Matter. 2016 Jul 28;12(34):7159–65. doi: 10.1039/c6sm01329e (PMC5322467; doi:10.1039/c6sm01329e)
Supplement: Supplementary file 1 [file SM-012-C6SM01329E-s001.pdf]

Electronic Supplementary Material (ESI) for Soft Matter.
This journal is © The Royal Society of Chemistry 2016

SUPPLEMENTARY INFORMATION

Hydrogen bonding asymmetric star-shape derivative of bile acid leads to supramolecular fibrillar aggregates that wrap into micrometer spheres

Teemu T. T. Myllymäki,[a] Nonappa,*[a] Hongjun Yang,[b] Ville Liljeström,[a] Mauri A. Kostiainen,[c] Jani-Markus Malho,[a] X. X. Zhu,[b] Olli Ikkala*[a]

[a] Department of Applied Physics, Aalto University, P.O. Box 15100, FI-00076 AALTO, Finland

E-mail: nonappa@aalto.fi, olli.ikkala@aalto.fi

[b] Department of Chemistry, Université de Montréal, C.P. 6128, Succursale Centre-ville, Montréal, QC H3C 3J7, Canada

[c] Department of Biotechnology and Chemical technology, Aalto University, P.O. Box 16100, FI-00076 AALTO, Finland

1. Measurements and instrumentation

1.1. Transmission Electron Microscopy (TEM)

TEM images were collected using FEI Tecnai G2 operated at 120 kV in bright field mode. TEM samples were prepared by dropcasting 1 mg/mL dispersion (5 µL) of the sample on Quantifoil R 2/1 Holey carbon film (Cu 200 Mesh) and the excess solvent was blotted with filter paper and let to evaporate to dry.

1.2. SerialEM and Electron Tomography (ET) Reconstruction

Tilt series for electron tomography (ET) reconstruction was acquired using the JEM 3200FSC. TEM samples were prepared by first depositing 3–5 µL of sample solution on carbon only 400 hexagonal mesh copper grids. The specimen temperature was maintained at -187 °C. For image alignment purposes, the TEM grids were dipped in 11-mercapto-1-undecanol ligand capped gold particle solution (diameter 3–5 nm) before sample deposition.[1] Electron tomographic tilt series were acquired with the SerialEM-software package.[2] Samples were tilted between ± 69 ° angles with 3 ° increment steps. Prealignment, fine alignment and the cropping of the tilt series were done with IMOD.[3] The images were binned twice for fibers and four times for spheres to reduce noise and computation time. Maximum entropy

method (MEM) reconstruction scheme was carried out with custom made program on Mac or Linux cluster with regularization parameter value of λ=1.0e^{-3}

1.3. Cryo-TEM

Cryogenic transmission electron microscopy (Cryo-TEM) imaging was done using Jeol 3200FSC cryo-transmission electron microscope at 300 kV in bright field mode. The images were acquired via an Omega-type Zero-loss energy filter and captured with Gatan Ultrascan 4000 CCD camera. The microscope was cooled down with liquid nitrogen and the sample temperature was maintained at -187 °C throughout the imaging. 3 µL of the sample dispersion was transferred on a Quantifoil R 2/1 Holey carbon (Cu 200 Mesh) grid in 100 % humidity. Excess amount of sample was blotted away with filter paper for 3x3 s and the samples were plunged into -170 °C ethane/propane (1/1 v/v) mixture using Fei Vitrobot Mk3. The vitrified samples were cryo-transferred to the microscope for imaging.

1.4. Scanning Electron Microscopy (SEM)

SEM imaging was performed on a Zeiss Sigma VP microscope at 2 kV voltage. A drop of dispersion (10 µL) was added on a carbon tape fixed on sample stub and allowed to dry at ambient conditions for 18 h. The sample was subjected for sputter coating with Au/Pd under vacuum at 20 mA for 3 minutes prior to imaging.

1.5. Nuclear Magnetic Resonance (NMR) spectroscopy

The solution state NMR spectra (^{1}H and ^{13}C NMR) were recorded on a Bruker Avance 400 MHz spectrometer equipped with 5 mm probe operating at 400.13 MHz for ^{1}H and 100.62 MHz for ^{13}C, respectively. The measurements were performed using deuterated chloroform ($CDCl_3$ 99.8 % atom D) or dimethyl sulfoxide (DMSO-d_6 99.96 % atom D) as the solvent and the chemical shifts referenced to trace of $CHCl_3$ (7.26 ppm) or DMSO-d_5 (2.50 ppm). In ^{1}H NMR measurements, number of scans was 64, a π/2 pulse length of 6.0 µs, the flip angle 30°, with recycle delay 2 s, and 64 K data points in time domain was collected which was zero filled to 128 K prior to FT. In ^{13}C NMR measurements, number of scans was 1024 and the spectra were processed with line broadening of 1 Hz before fourier transform and the chemical shifts were referenced to $CHCl_3$ triplet (76.00 ppm) or DMSO-d_5 sextet (39.50 ppm). Sample was dissolved 5 mg/mL for ^{1}H NMR measurements and 10 mg/mL for ^{13}C NMR studies.

1.6. Fourier Transform Infra-Red Spectroscopy (FT-IR)

FT-IR spectra of solid samples were measured on a Nicolet 380 spectrometer (Thermo Fisher Scientific) in transmission mode with an ATR (diamond), 2 cm^{-1} resolution and averaged over 64 scans.

1.7. Small-Angle X-ray Scattering (SAXS)

Samples with varying DMSO/H_2O ratio were prepared by adding ultrapure water to CA(AGE_6-C_6H_{12}-UPy$)_4$ dissolved in DMSO. Addition of water caused formation of larger assemblies which was visually observed as a milky appearance of the samples. The samples were sealed between Kapton foils during the SAXS measurement. The sample environment was evacuated to reduce scattering from air. The SAXS was measured using a rotating anode Bruker Microstar microfocus X-ray source (Cu Kα radiation, λ = 1.54 Å). The beam was monochromated and focused by a Montel multilayer focusing monochromator (Incoatec). The X-ray beam was further collimated by a set of four slits (JJ X-ray). The size of the X-ray beam at the sample position was < 1mm. The scattered intensity was collected using a Hi-Star 2D area detector (Bruker). Sample-to-detector distance was 1.59 m, and silver behenate standard sample was used for calibration of the length of the scattering vector q. One-dimensional SAXS data were obtained by azimuthally averaging the 2D scattering data. The magnitude of the scattering vector q is given by $q = 4\pi \sin\theta / \lambda$, where 2θ is the scattering angle.

1.8. DLS measurements

DLS data was achieved with Zetasizer Nano ZS instrument (model no. ZEN3600) with two different concentrations: 1 mg/mL and 0.1 mg/mL of sample in the used solvent mixtures.

1.9. Size Exclusion Chromatography (SEC)

SEC measurements were performed with a Waters chromatograph equipped with three Styragel columns (HR2, HR4, HR6) and a Waters 410 differential refractometer (Waters Instruments, Rochester, MN). A solution of *N,N*-dimethylformamide (DMF) and LiCl (1 mg/mL) was used as an eluent with a flow rate of 0.8 mL/min. Polystyrene standards (PSS Polymer Standards Service GmbH) were used for the calibration.

2. Experimental

2.1. General methods and materials

All the syntheses were performed in clean and oven dried (120 ºC) glassware. Reagents and solvents were purchased from commercial sources. 1,6-diisocyanatohexane (98 % purity), dibutyltin dilaurate (DBTDL, 95 % purity) were obtained from Sigma Aldrich and used without further purification. Technical/Analytical grade solvents (chloroform, *N,N*-dimethylformamide, dimethyl sulfoxide) were purchased from Sigma-Aldrich. The allyl glycidyl ether derivatized cholic acid, denoted $CA(AGE_6)_4$, (Fig. S1), was prepared and characterized by previously published method.[4] Deuterated solvents ($CDCl_3$ and DMSO-d_6) were obtained from Sigma-Aldrich and used as received.

2.1. Dialysis

Dialyses were performed using Spectra/Por 7 Standard Regenerated Cellulose tubing (Mw cut-off 2000 Da). Ultrapure deionized water with resistivity of 18 MΩ/cm was used. In a typical dialysis procedure, the sample was dissolved in DMSO at 1 mL/mg concentration. 1 mL of the solution was transferred to a dialysis tube, air was extracted from the tube and it was sealed tightly, but with extra tube length so that the volume inside the tube can increase without increase in pressure.

The dialysis tube containing the sample dissolved in DMSO was immersed in DMSO (500 ml). To this DMSO bath, water was added to achieve different compositions of DMSO and water. To achieve lower DMSO amounts in water, part of the solvent mixture was poured off and water was added, until there was only water left. Samples for different measurements were taken during the dialysis, after allowing the solvent exchange to happen inside the tube.

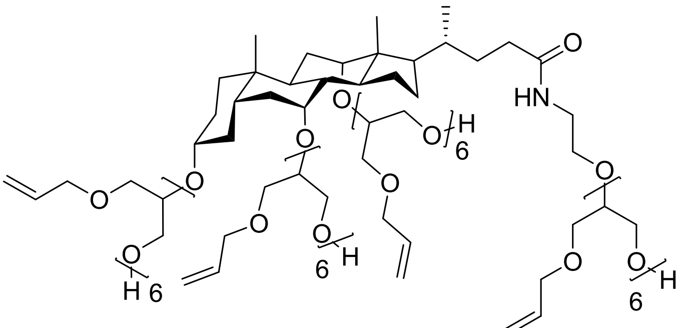

1

Fig. S1 Cholic acid with four allyl glycidyl ether branches, each six monomers long (**1**), abbreviated $CA(AGE_6)_4$.

2.2. Synthesis of Ureidopyrimidinone isocyanate (UPy-NCO)

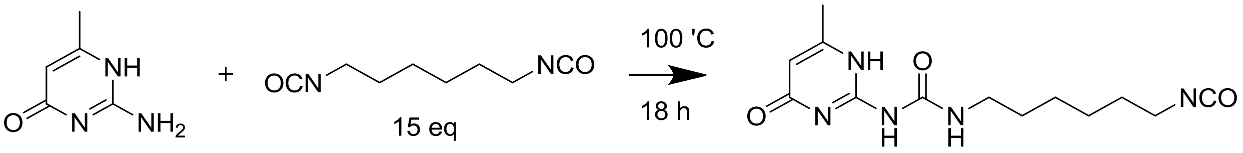

2 **3** **4**

Fig. S2 Synthesis of UPy-NCO

2-Ureido-4[1H]pyrimidinone with isocyanate hexyl linker (UPy-NCO) **4** was prepared by adding 1.6 g of 2-amino-4-hydroxy-6-methylpyrimidine **2** and 18 mL 1,6-diisocyanatohexane **3** in a dry 250 mL 2-neck round-bottom flask equipped with stirring, condenser and nitrogen flow. After heating 19 h at 100 °C in an oil bath, the mixture was let to cool to room temperature. Mixture was transferred to 50 mL hexane, filtered with Büchner funnel and washed with 100 mL hexane. Clear white crystals were placed to vacuum oven at 50 °C to dry overnight. Yield: 3.33 g (84 %).

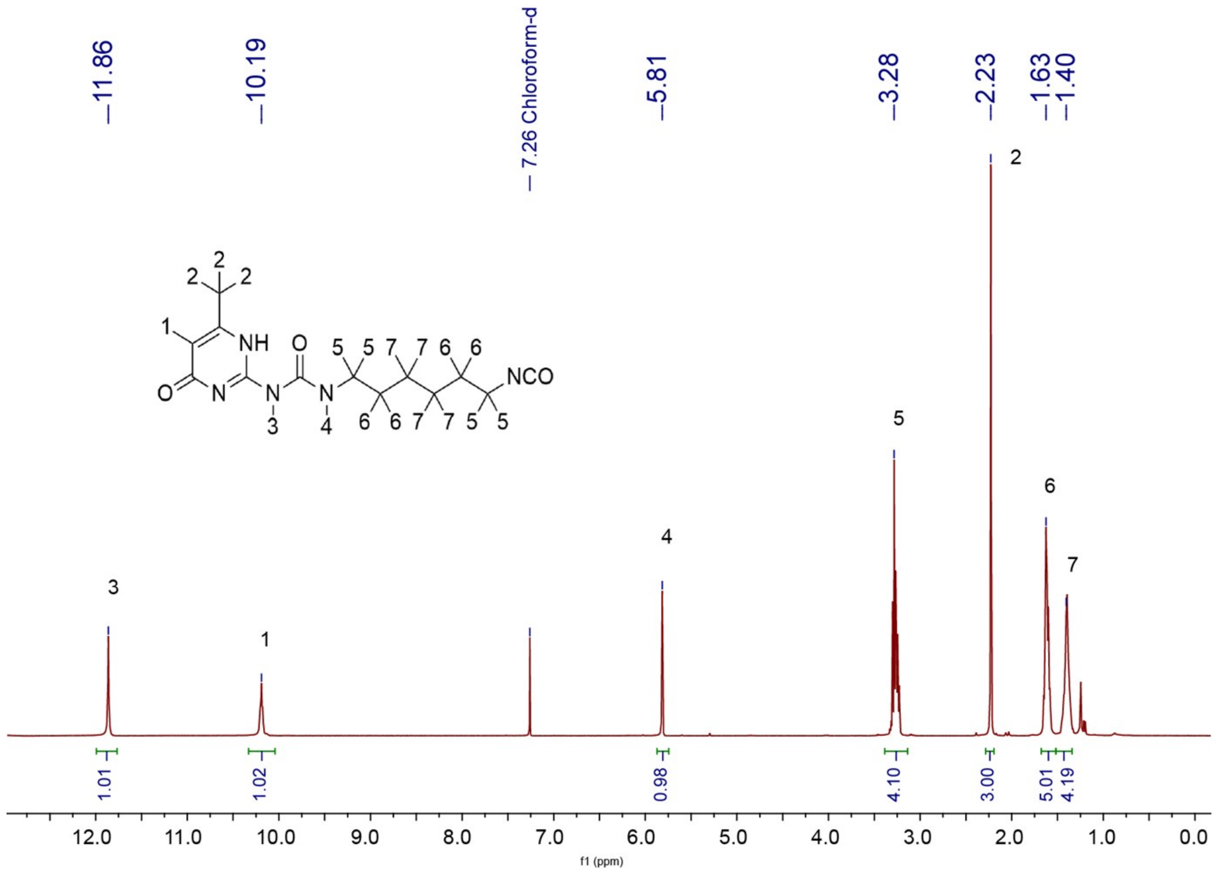

Fig. S3 The ^{1}H NMR spectrum (400 MHz, $CDCl_3$) of UPy-NCO.

^{1}H NMR (400 MHz, $CDCl_3$, Fig. S3): δ 11.86 (s, 1H), 10.19 (s, 1H), 5.81 (s, 1H), 3.28 (m, 4H), 2.23 (s, 3H), 1.63 (m, 4H), 1.40 (m, 4H).

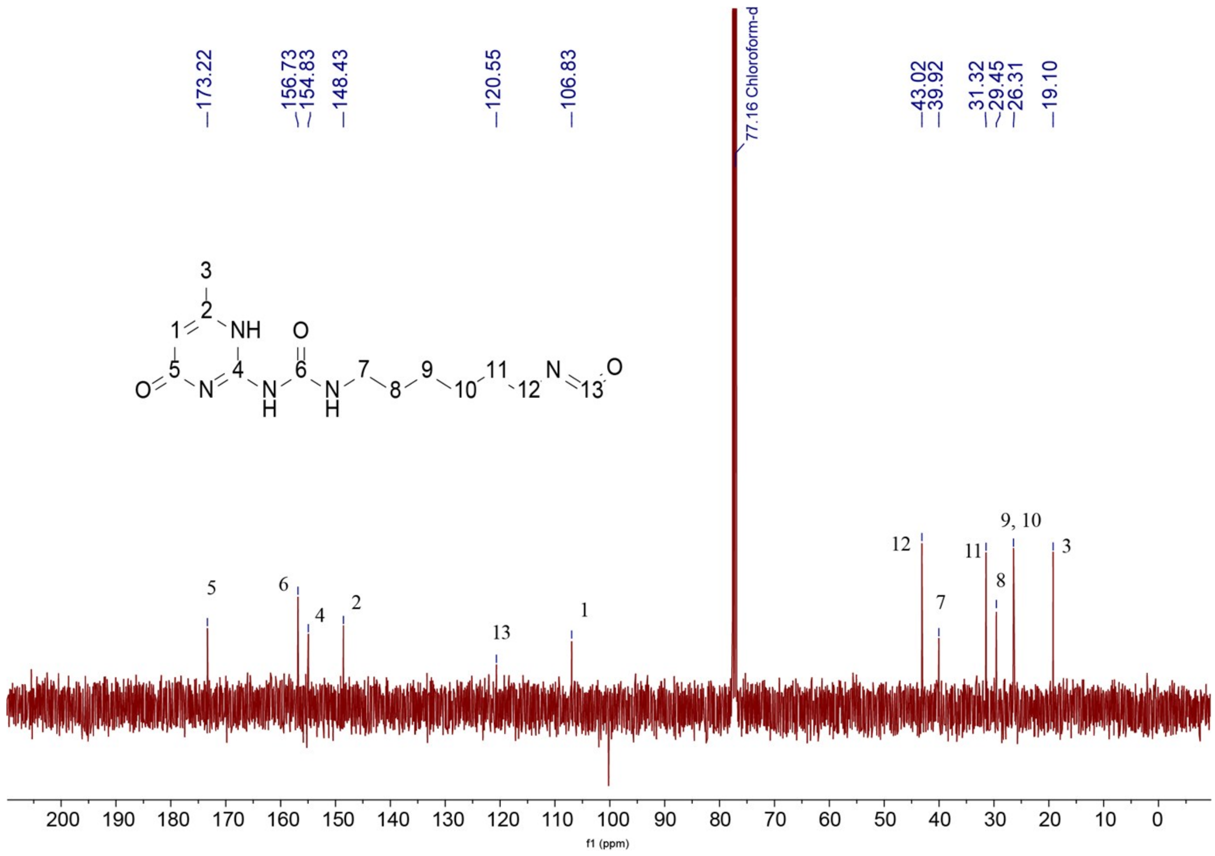

Fig. S4 The ^{13}C NMR spectrum (400 MHz, $CDCl_3$) of UPy-NCO

^{13}C NMR (100 MHz, $CDCl_3$, Fig. S4): δ 173.22, 156.73, 154.83, 148.43, 120.55, 106.83, 77.16, 43.02, 39.92, 31.32, 29.45, 26.31, 19.10.

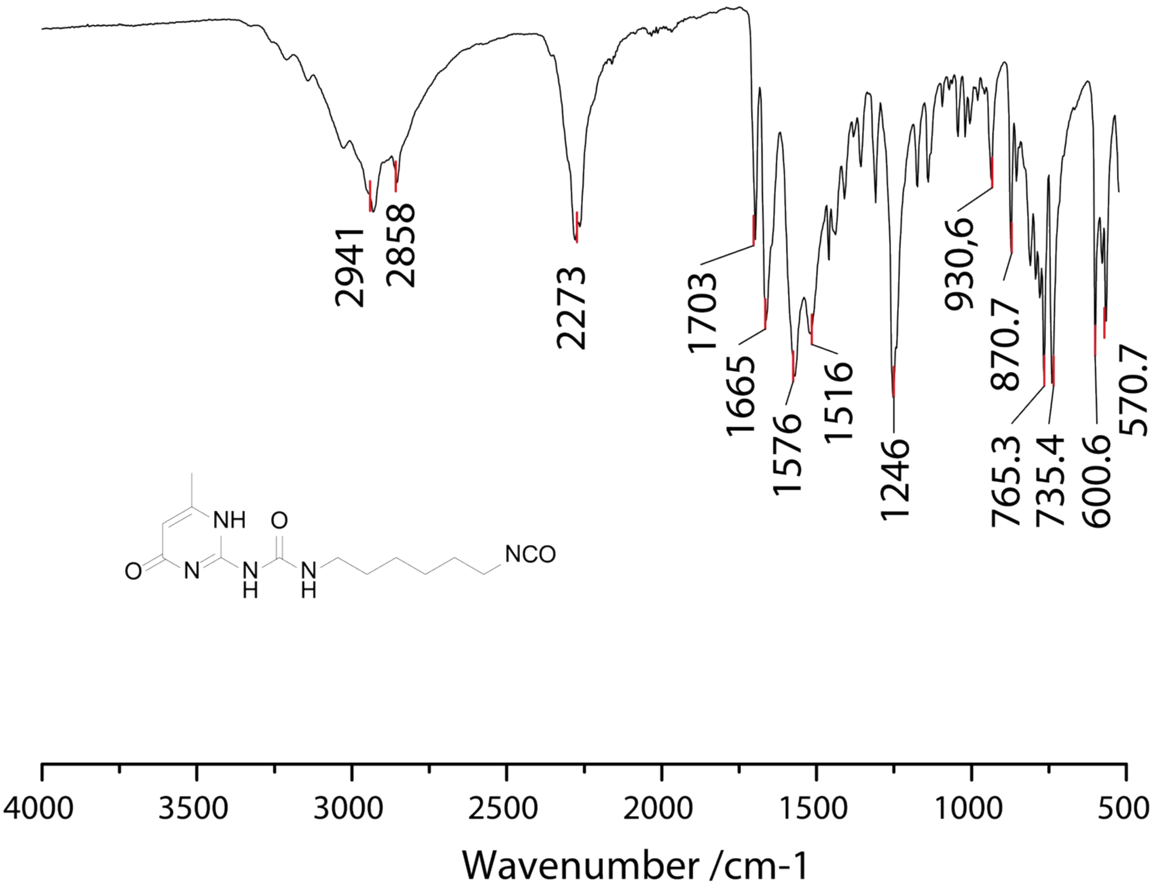

Fig. S5 FT-IR spectrum of UPy-NCO (**4**).

FT-IR (ATR, Fig. S5) υ cm^{-1}: 3308, 2933, 2858, 2258, 1688, 1621, 1516, 1440, 1358, 1253, 1208, 1095, 862.9, 758.1, 660.5.

2.3. Synthesis of CA(AGE_6-C_6H_{12}-UPy)$_4$

The asymmetric star-like cholic acid derivative with hydrogen bonding domains (Fig. S6, **5)** were prepared by adding UPy-NCO (**4**) (0.087 g) to a dry 10 mL round-bottom flask. Also 5 mL of dry chloroform, 100 µL dibutyltin dilaurate (DBTDL) and 0.2 g of **1** were added. The flask was equipped with a condenser and a $CaCl_2$ tube. The mixture was stirred and refluxed for 24 h. After cooling down the mixture was transferred to 30 mL of hexane and centrifuged (3000 G, 10 min). The resulting solids were freeze-dried. Yield was 0.27 g.

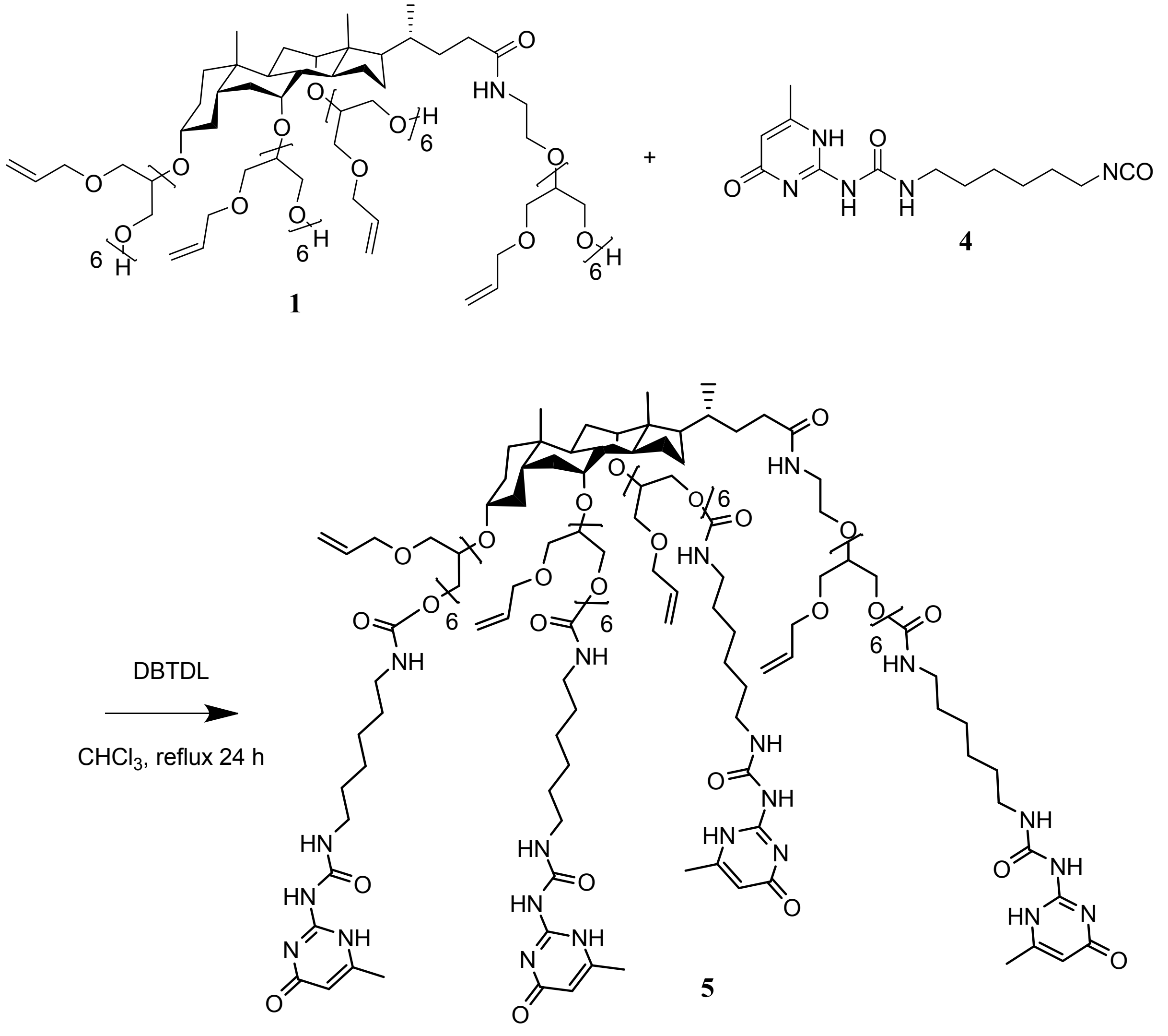

Fig. S6 Synthesis of asymmetric star-like cholic acid derivative with hydrogen bonding domains CA(AGE_6-C_6H_{12}-UPy)$_4$.

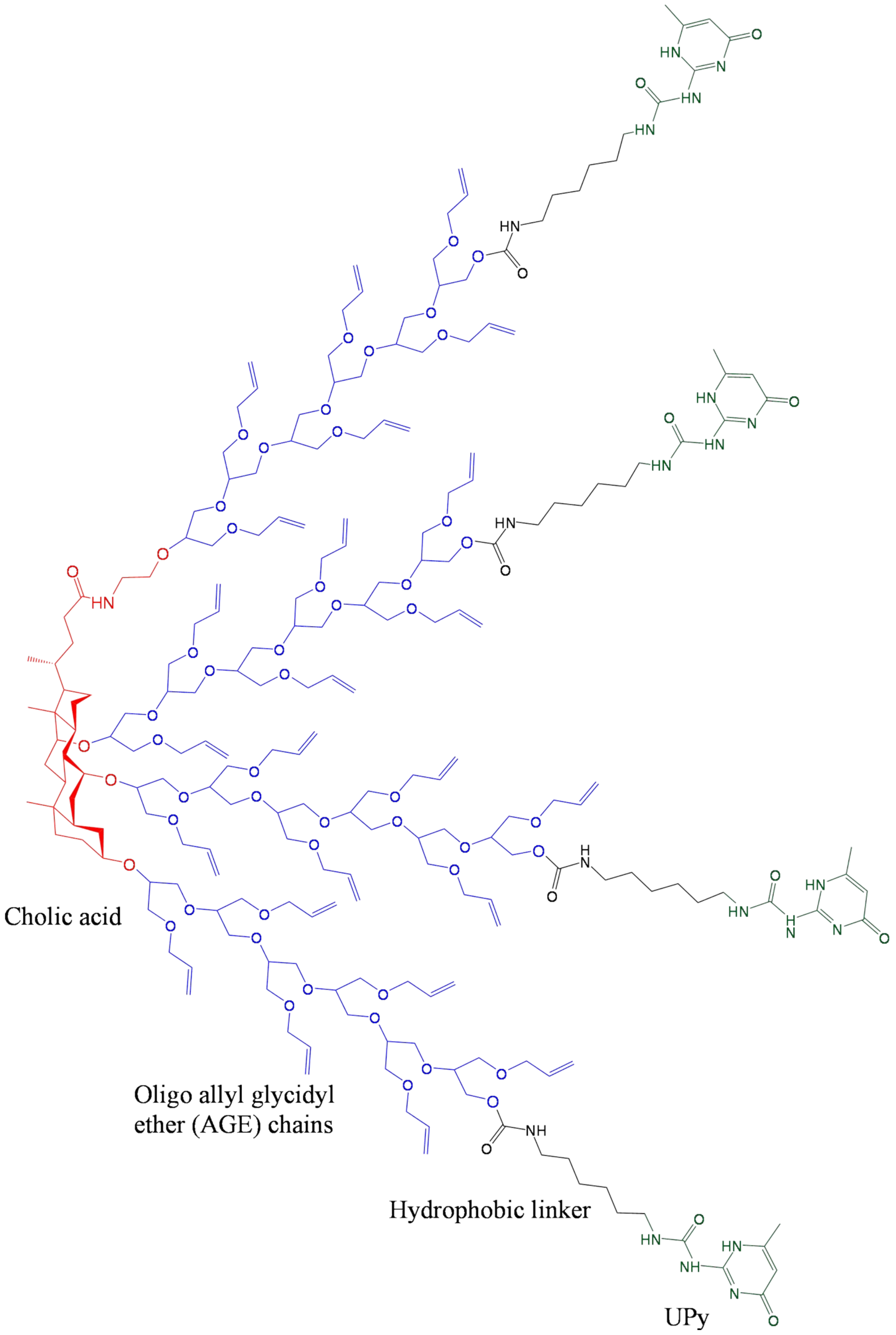

Fig. S7 Asymmetric star-like cholic acid derivative $CA(AGE_6\text{-}C_6H_{12}\text{-}UPy)_4$.

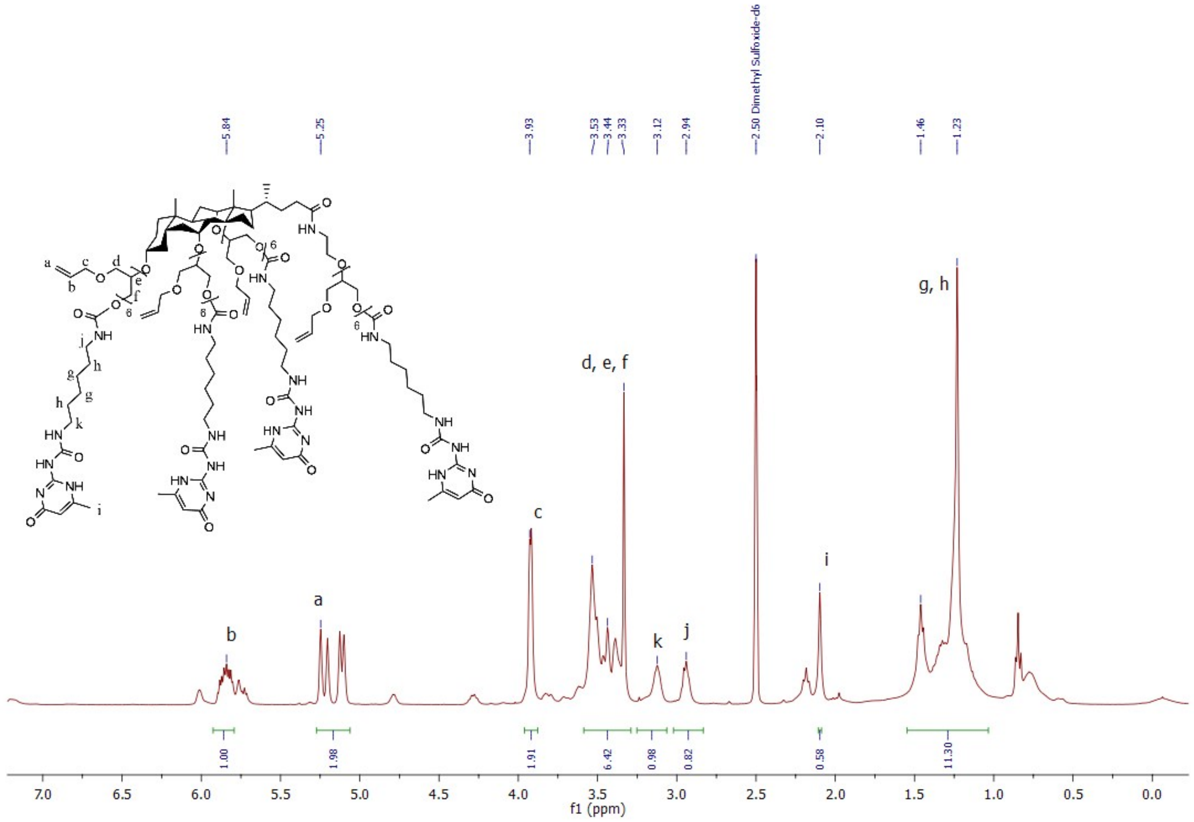

Fig. S8 The ^{1}H NMR spectrum (400 MHz, DMSO-d_6) of CA(AGE_6-C_6H_{12}-UPy)$_4$. Letters in spectrum corresponds to protons attached to carbons labelled with the letter.

^{1}H NMR (400 MHz, DMSO-d_6, Fig. S8) δ 1.24, 1.47, 2.10, 2.95, 3.13, 3.34, 3.54, 3.93, 5.25, 5.85.

The amount of UPy per polymer chain according to ^{1}H NMR integrals:

(a) corresponds to 48 protons with integral 1.98

(i) corresponds to 12 protons with integral 0.65

Integral per proton according to a: 1.98/48=0.04125

Integral assumed for i: 12*0.04125=0.495 (found 0.58)

According to integrals the UPy -CH_3 (i) either relaxes faster than the allyl protons in the polymer chain (a), resulting in higher integral, or there is a small excess of UPy compared to the polymer chains.

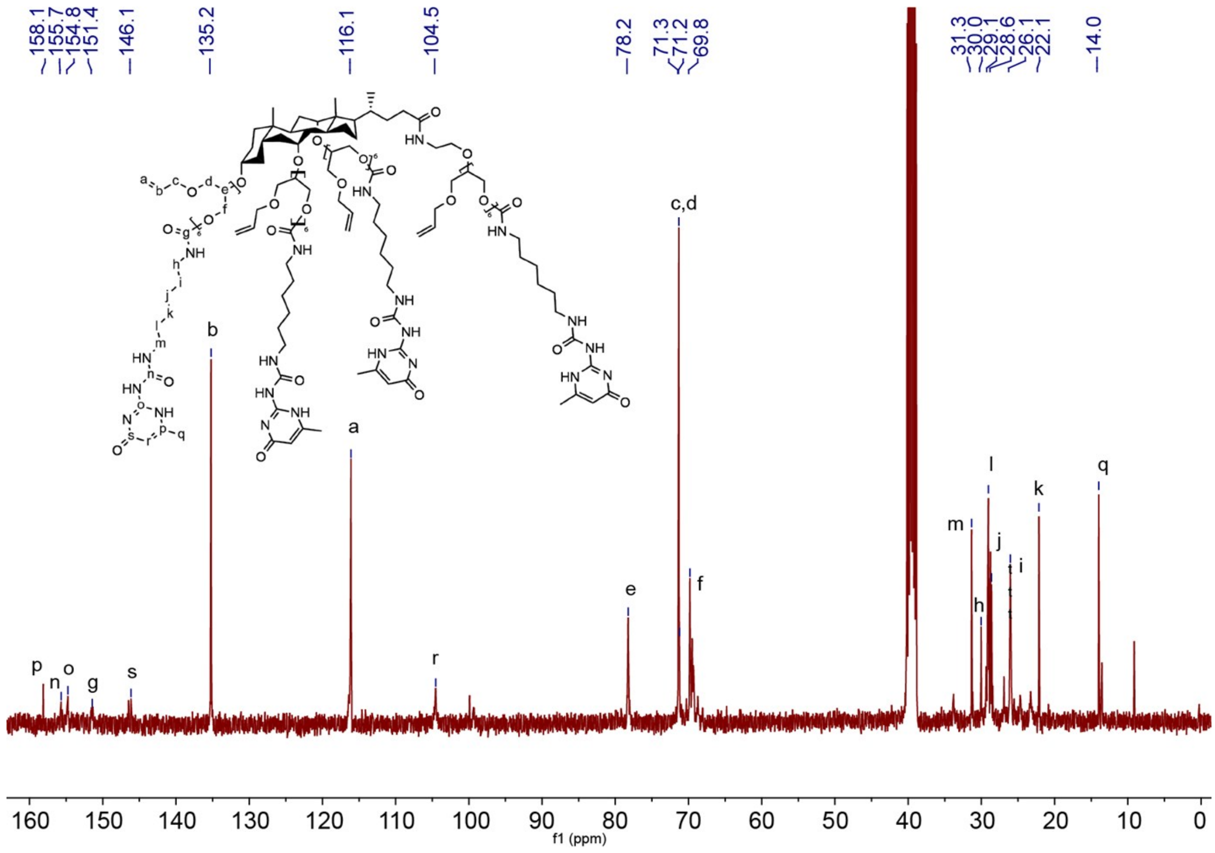

Fig. S9 The ^{13}C NMR spectrum (100 MHz, DMSO-d_6) of CA(AGE$_6$-C_6H_{12}-UPy)$_4$.

^{13}C NMR (100 MHz, DMSO-*d*$_6$, Fig. S9) δ 14.0, 22.1, 26.1, 28.6, 29.1, 30.0, 31.3, 69.8, 71.2, 71.3, 78.2, 104.5, 116.1, 135.2, 146.1, 151.4, 154.8, 155.7, 158.1.

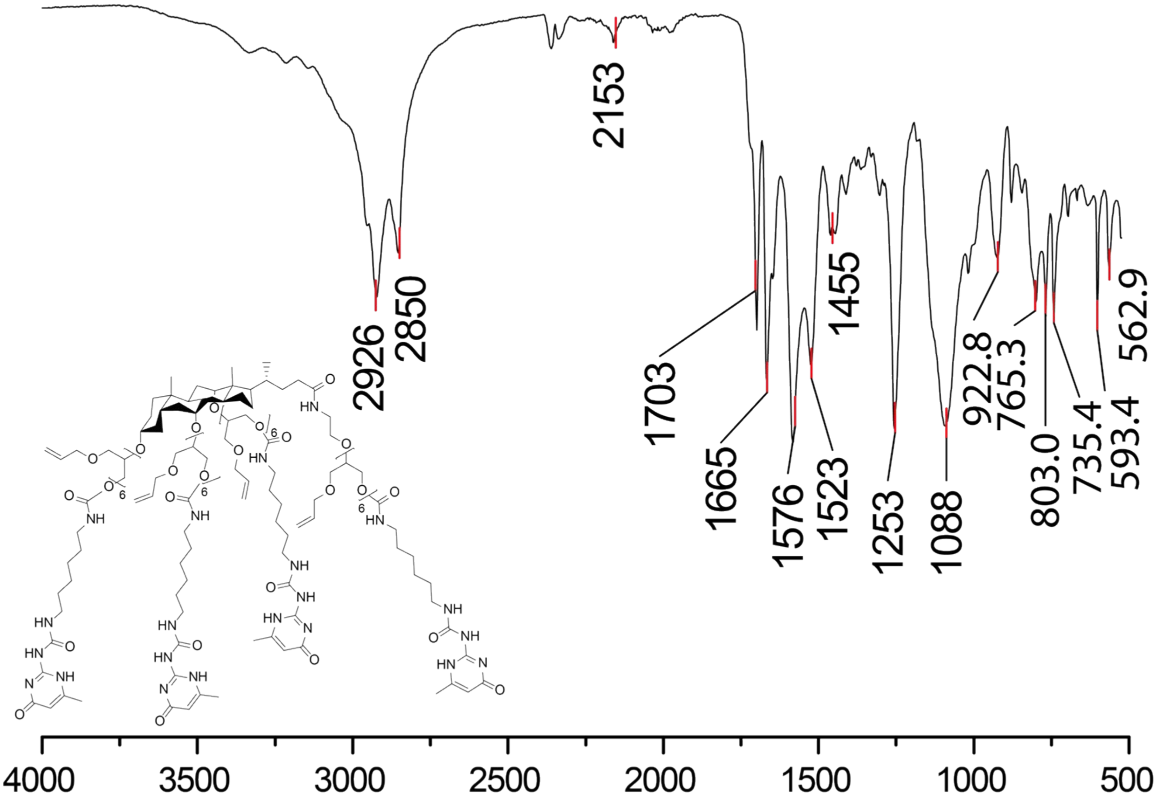

Fig. S10 FT-IR spectrum of CA(AGE$_6$-C$_6$H$_{12}$-UPy)$_4$.

FT-IR (ATR, Fig. S10) υ cm^{-1}: 2926, 2850, 2153, 1703, 1665, 1576, 1455, 1253, 1088, 922.8, 765.3, 803.0, 735.4, 593.4, 562.9.

2.1. Synthesis of CA(AGE$_6$-C$_6$H$_{13}$)$_4$

CA(AGE$_6$-C$_6$H$_{13}$)$_4$ (Fig. S11, **7**) was prepared by adding 39.3 mg hexyl isocyanate (**6**) to a dry 10 mL round-bottom flask. Also 5 mL of dry chloroform, 100 µL dibutyltin dilaurate (DBTDL) and 0.2 g of **1** were added. The flask was equipped with a condenser and a $CaCl_2$ tube. The mixture was stirred and refluxed for 24 h. After the reaction was finished, 1 mL of water was added and the mixture was dried with rotary evaporator. The product was dissolved in hexane and purified with dialysis against hexane. Yield was 0.17g.

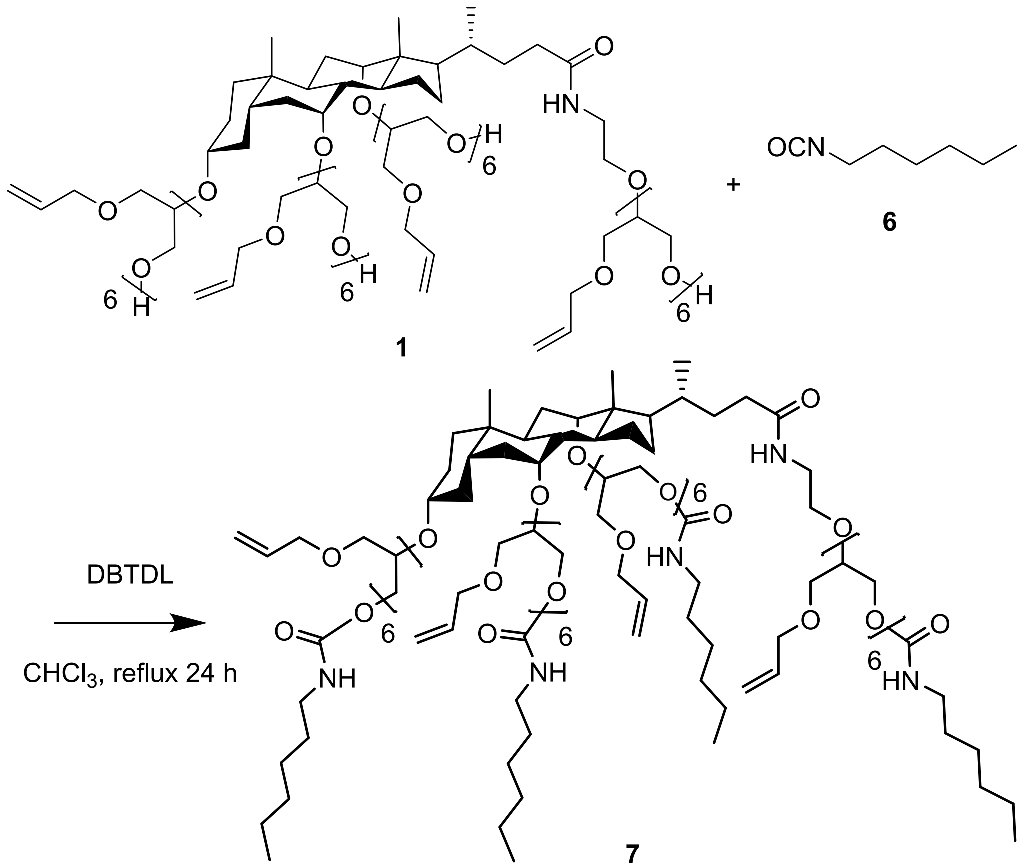

Fig. S11 Synthesis of CA(AGE_6-C_6H_{13})$_4$.

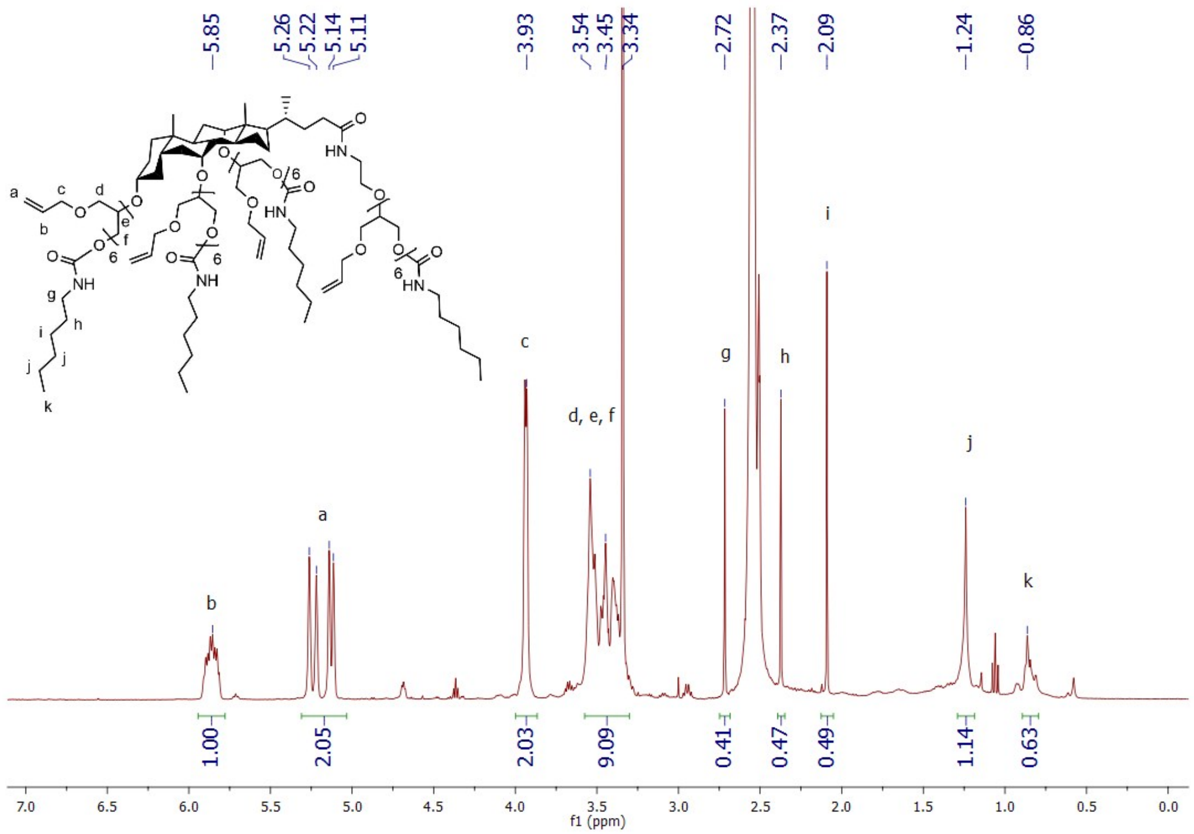

Fig. S12 The ^{1}H NMR spectrum (400 MHz, DMSO-d$_6$) of CA(AGE$_6$-C$_6$H$_{13}$)$_4$.

^{1}H NMR (400 MHz, DMSO-*d$_6$*, Fig. S12) δ 5.85, 5.25, 5.22, 5.14, 5.11, 3.93, 3.54, 3.45, 3.34, 2.27, 2.37, 2.09, 1.24, 0.86.

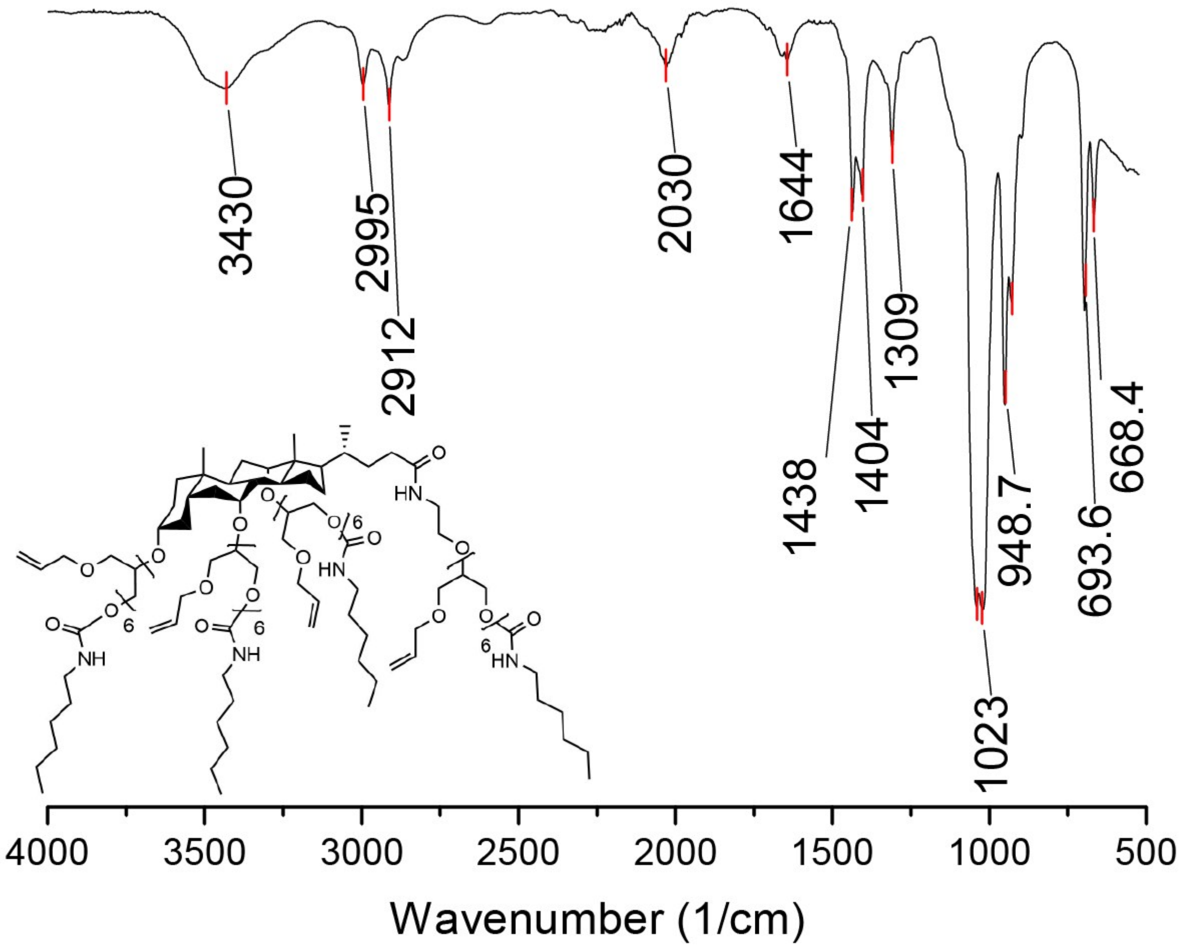

Fig. S13 FT-IR spectrum of CA(AGE_6-C_6H_{13})$_4$.

FT-IR (ATR, Fig. S13) υ cm^{-1}: 3430, 2995, 2912, 2030, 1644, 1438, 1404, 1309, 1023, 948.7, 693.6, 668.4.

3. DLS measurements

Table S1 The hydrodynamic diameters for $CA(AGE_6\text{-}C_6H_{12}\text{-}UPy)_4$ and $CA(AGE_6)_4$ in a low 0.1 mg/mL concentration at different compositions of DMSO/water mixtures. The values in parentheses correspond to those where the classic CONTIN-analysis is expected to fail, due to non-spherical particles.

	Diameter (pk mean int /nm)	
Amount of water in DMSO (%)	$CA(AGE_6\text{-}C_6H_{12}\text{-}UPy)_4$	$CA(AGE_6)_4$
0	11.55	9.2
20	252.7	204.8
40	(712.4)	(166.8)
60	(138.8)	(96.94)
80	(306.5)	(58.39)
100	888.4	34.67

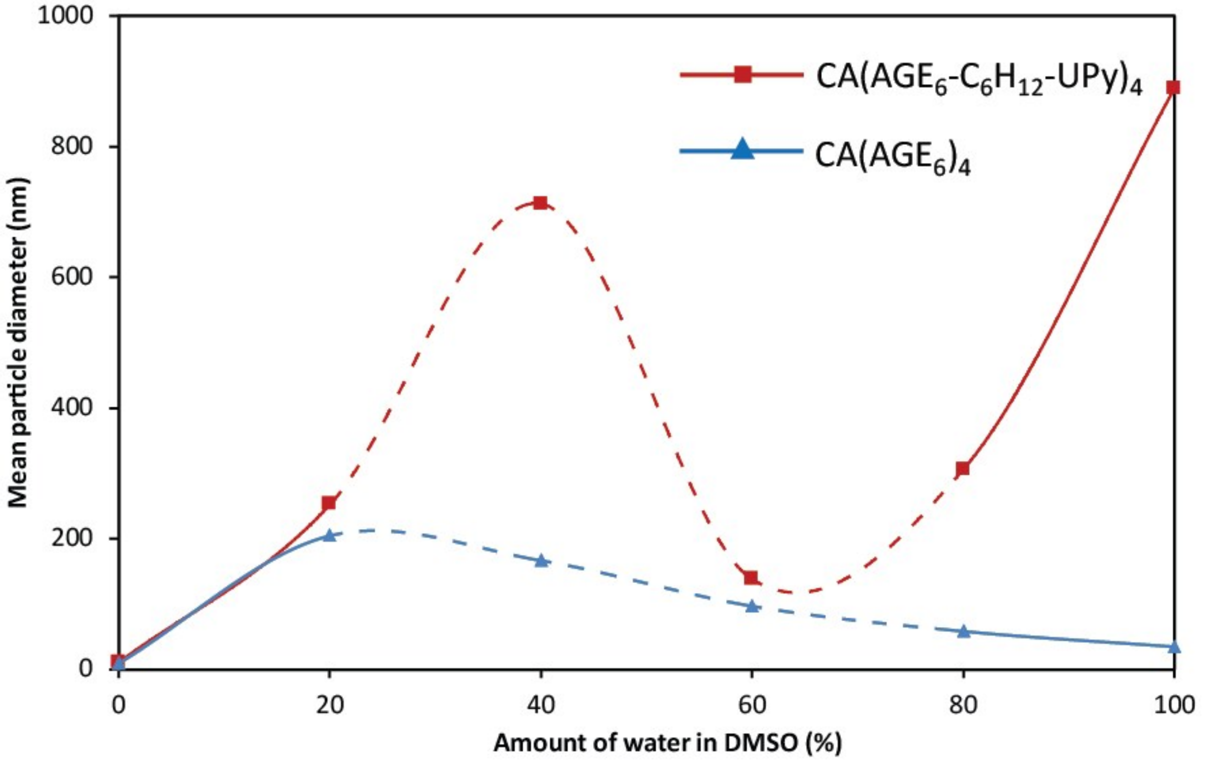

Fig. S14 Hydrodynamic diameter of $CA(AGE_6\text{-}C_6H_{12}\text{-}UPy)_4$ and $CA(AGE_6)_4$ in different compositions of DMSO/water mixtures in 0.1 mg/ml concentration, measured by DLS. The lines serves guide to the eye. The approximate data range, where the classic CONTIN-analysis is not valid due to co-existence of fibers and spheres is marked by dotted lines.

It needs to be noted that fixed angle DLS measurements are suitable only for spherical particles in a solvent. Since CA(AGE$_6$-C$_6$H$_{12}$-UPy)$_4$ self-assembles in various structures that are far from spherical, as resolved in TEM studies, in DMSO-water mixtures the data needs to be considered only qualitatively. On the other hand, the particles in pure DMSO, and in water, in the case of CA(AGE$_6$-C$_6$H$_{12}$-UPy)$_4$, are roughly spherical and therefore the presented data for hydrodynamic range from Zetasizer Nano ZS is valid.

4. SAXS measurements

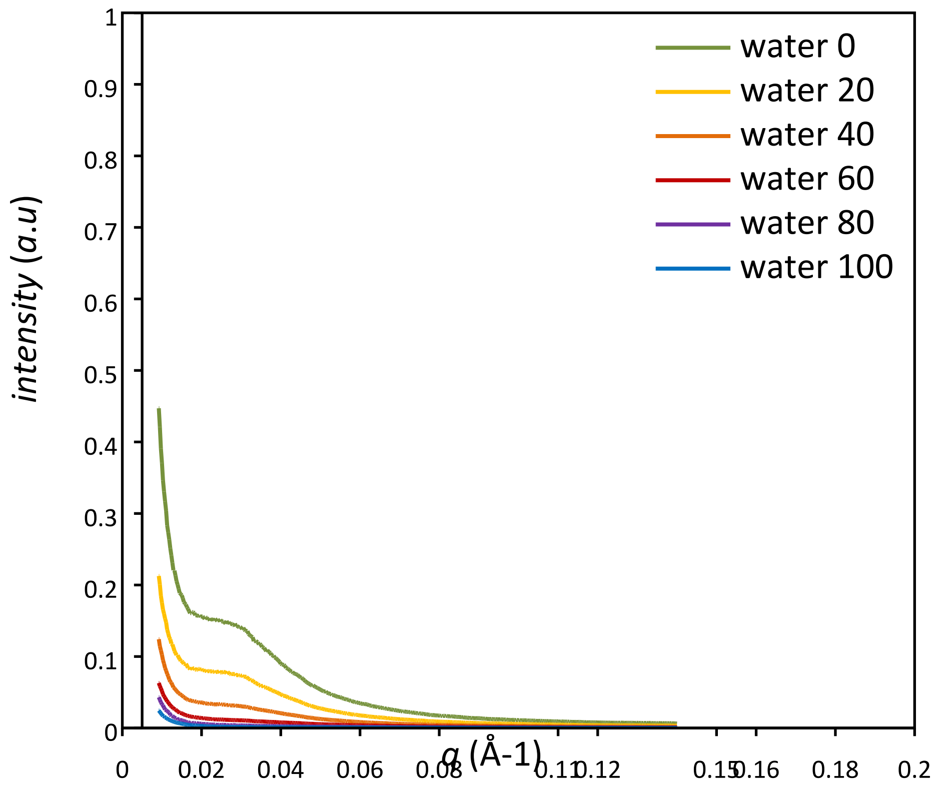

Fig. S15 SAXS measurements of CA(AGE$_6$-C$_6$H$_{12}$-UPy)$_4$ in different DMSO-water mixtures upon dialysis from DMSO to water (the labels indicate the fraction of water in water/DMSO mixtures).

SAXS of CA(AGE$_6$-C$_6$H$_{12}$-UPy)$_4$, as dissolved in DMSO, shows a broad correlation peak at the scattering vector magnitude q ~ 0.035-0.045 Å^{-1} corresponding to structural features of 14-18 nm, and is interpreted as the form factor of micelles of CA(AGE$_6$-C$_6$H$_{12}$-UPy)$_4$. For

samples with increasing amount of water the form factor gradually disappears, which agrees that the structurally less-defined larger aggregates start forming.

5. SEC measurements

Here we illustrate the challenges of molar mass measurements of CA(AGE$_6$-C$_6$H$_{12}$-UPy)$_4$ due to UPy induced aggregation (Fig. S16).

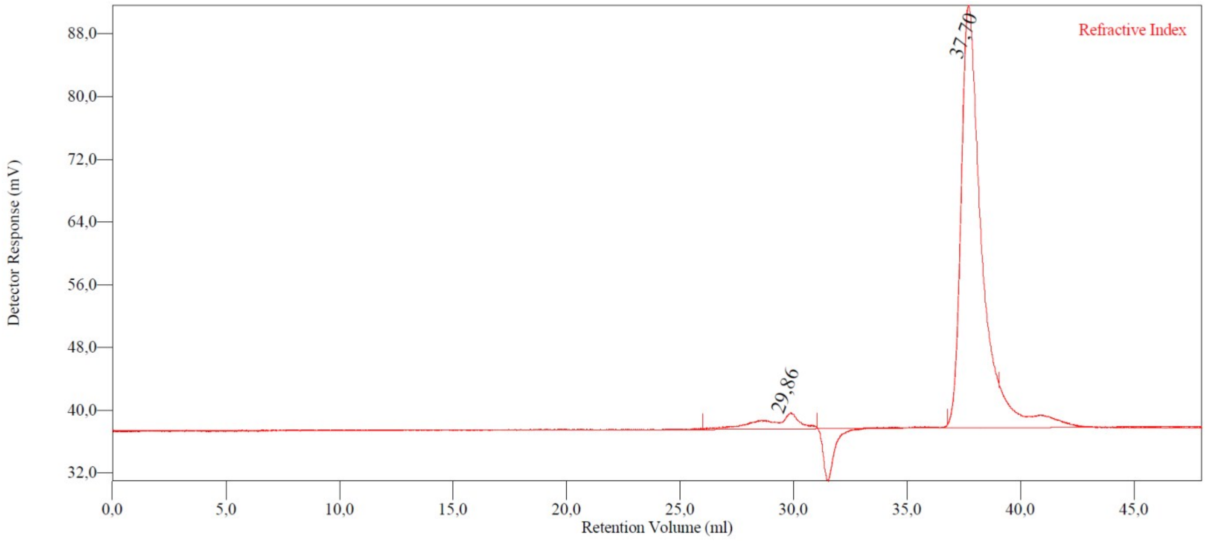

Fig. S16 SEC in DMF with refractive index detector of CA(AGE$_6$-C$_6$H$_{12}$-UPy)$_4$.

The SEC measurement gave Mn 9522 Da (Mw 12851 Da) for molar mass of CA(AGE$_6$-C$_6$H$_{12}$-UPy)$_4$. However, due to the incompatibility between the solvent (DMF) and the nonpolar cholic acid core, it is feasible that the molecules form aggregates or micelles in DMF, as we have shown in DMSO. Therefore SEC cannot resolve molar mass of single CA(AGE$_6$-C$_6$H$_{12}$-UPy)$_4$ molecule, but instead molar mass of aggregates formed in polar solvent such as DMF. We would like to acknowledge M.Sc. Sami-Pekka Hirvonen, A.I Virtasen aukio 1, 4th floor, P.O.Box 55, 00014 University of Helsinki for the measurements.

6. TEM measurements

6.1. Particle size analysis

Particles for analysis were picked by hand due to poor contrast in cryo-TEM measurements. The diameter of the particles were measured with Gatan Microraph software using contrast difference tool. Particles in same focus plane were used to prevent error due to over or under focus.

Table S2 Particle size analysis for $CA(AGE_6)_4$ and $CA(AGE_6\text{-}C_6H_{12}\text{-}UPy)_4$ in DMSO according to Cryo-TEM measurements.

Particle	$CA(AGE_6\text{-}C_6H_{12}\text{-}UPy)_4$ particle diameter (nm)	$CA(AGE_6)_4$ particle diameter (nm)
1	12.56	15.95
2	13.94	12.99
3	18.01	12.76
4	15.85	8.67
5	18.01	10.04
6	19.16	10.48
7	14.23	10.05
8	13.93	12.53
9	19.95	10.51
Average	16.2	11.6
Standard deviation	2.7	2.2

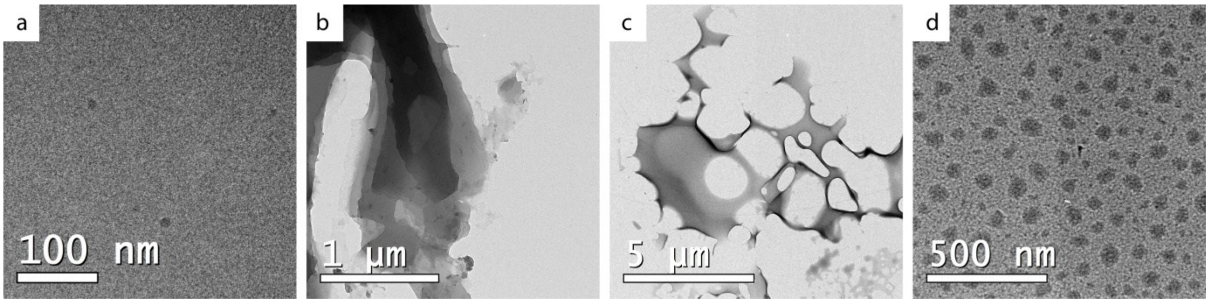

Fig. S17 TEM micrographs of $CA(AGE_6)_4$ molecules, i.e., without UPy modification in different DMSO/water mixtures. (a) Cryo-TEM image in DMSO. TEM images from samples dried from (b) DMSO/water 80/20 v/v, (c) DMSO/water 50/50 v/v, and (d) water.

6.2. Cryo-TEM of CA(AGE$_6$-C$_6$H$_{12}$-UPy)$_4$ in DMSO

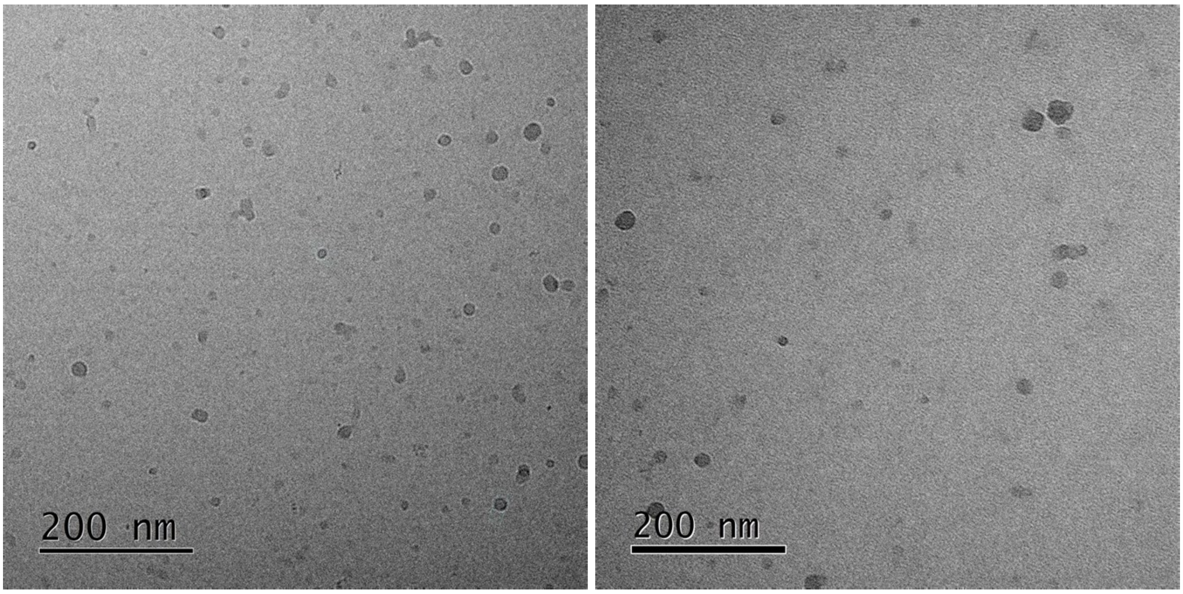

Fig. S18 Cryo-TEM images of CA(AGE$_6$-C$_6$H$_{12}$-UPy)$_4$ in DMSO

6.3. Cryo TEM of CA(AGE$_6$)$_4$ in DMSO

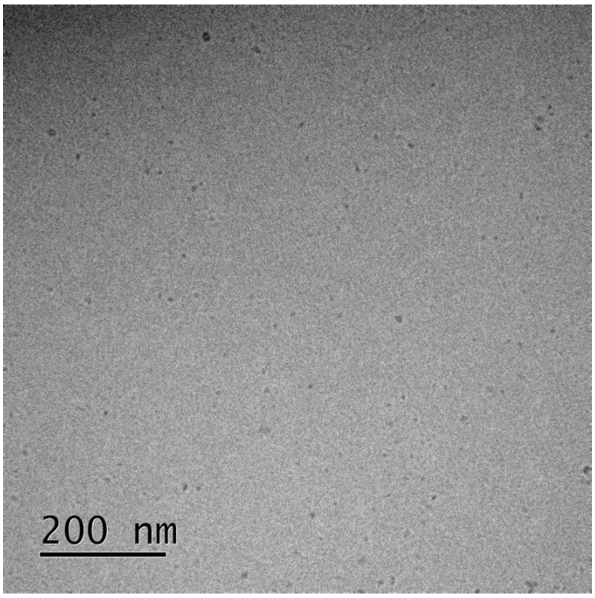

Fig. S19 Cryo-TEM of CA(AGE$_6$)$_4$ in DMSO

6.4. Conventional TEM measurements of $CA(AGE_6\text{-}C_6H_{12}\text{-}UPy)_4$ in DMSO, water and mixtures thereof

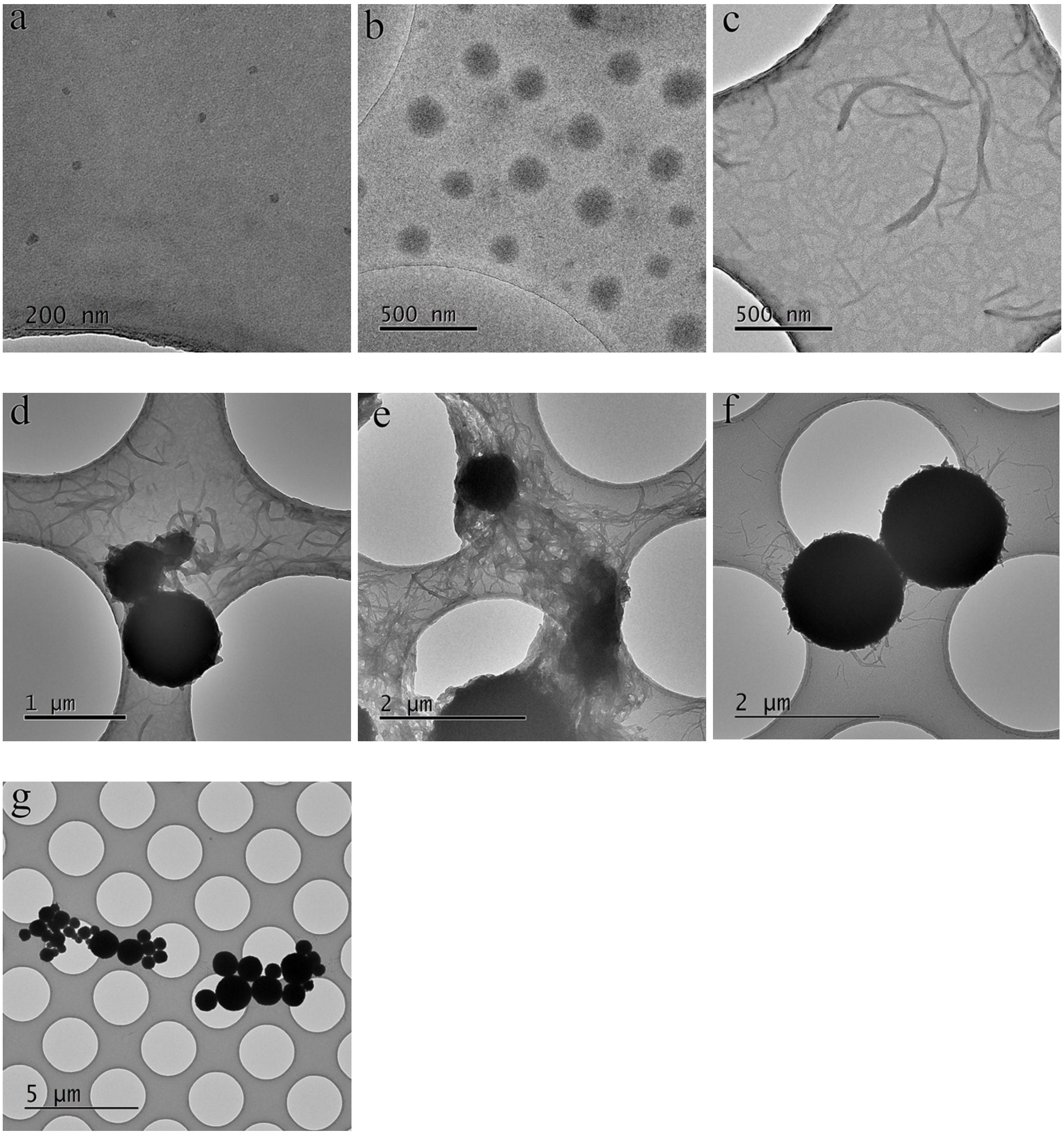

Fig. S20 Conventional TEM (without vitrification) of $CA(AGE_6\text{-}C_6H_{12}\text{-}UPy)_4$ in different solvent compositions. a) in DMSO. Note that this is a dried sample and aggregation may have occurred, hence larger particles compared to Cryo-TEM measurements are present. b) in DMSO/water 60/40 v/v. This is a borderline composition, showing small micellar aggregates and also onset of larger structures. c) in DMSO/water 50/50 v/v d) in DMSO/water 50/50 v/v e) in DMSO/water 50/50 v/v f) in water g) in water.

6.5. Conventional TEM measurements of $CA(AGE_6\text{-}C_6H_{13})_4$

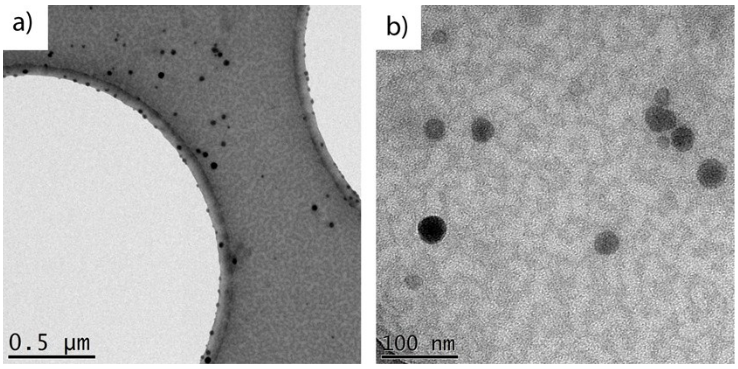

Fig. S21 Conventional TEM (without vitrification) of $CA(AGE_6\text{-}C_6H_{13})_4$ (**7**) after being dialyzed from DMSO to water.

7. TEM tomographies

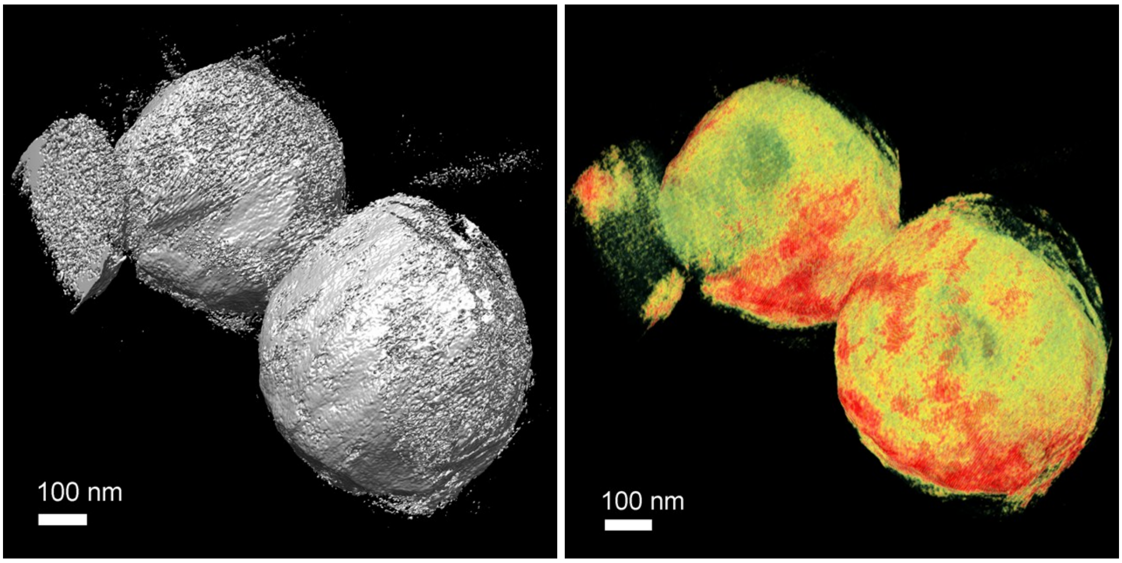

Fig. S22 (left) TEM tomography 3D reconstruction of two spheres. (right) TEM tomography 3D reconstruction of two spheres with density mapping.

8. SEM measurements

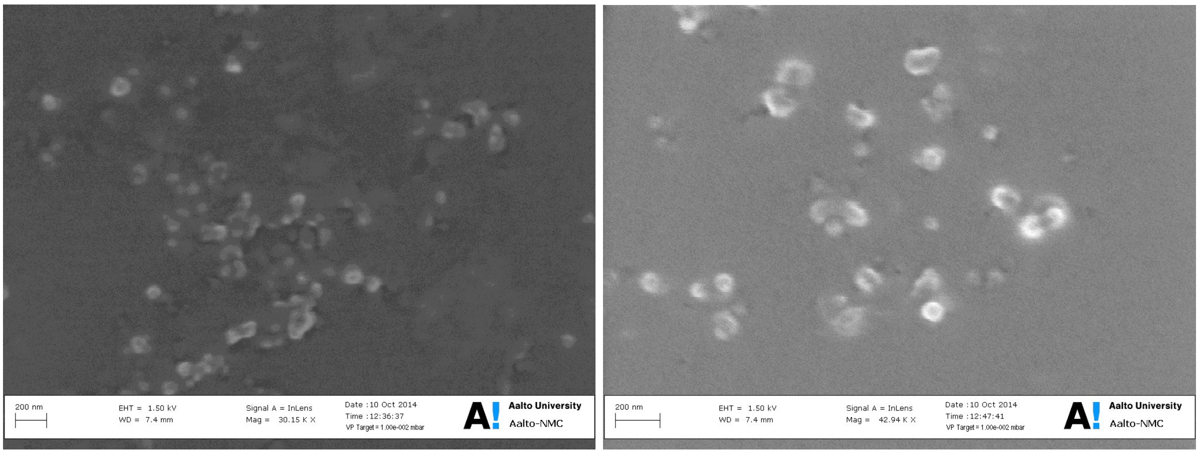

Fig. S23 SEM of CA(AGE_6-C_6H_{12}-UPy)$_4$ in DMSO

In DMSO, the particle sizes appear larger using SEM than using TEM. The reason for the different particle size might be in sample preparation, as the SEM samples were prepared by dropcasting 10 µL of solution on SEM stub followed by evaporation to dryness. During the evaporation the concentration of CA(AGE_6-C_6H_{12}-UPy)$_4$ gradually increases, potentially resulting changes in the self-assembly. Also, upon drying the DMSO-water mixtures, water evaporates first, resulting in concentrated DMSO. Therefore SEM studies are to be considered only qualitatively.

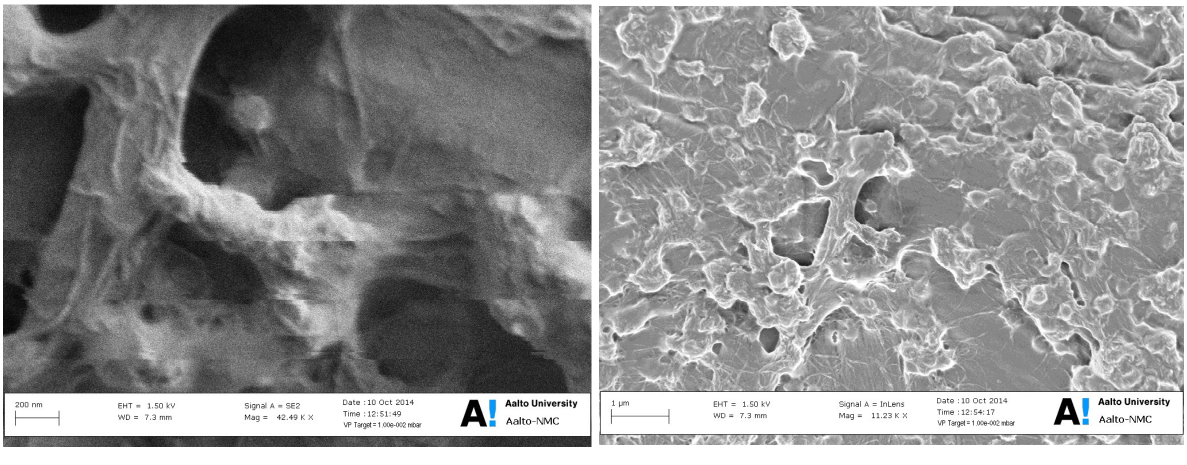

Fig. S24 SEM of CA(AGE_6-C_6H_{12}-UPy)$_4$ in DMSO/water 70/30 v/v

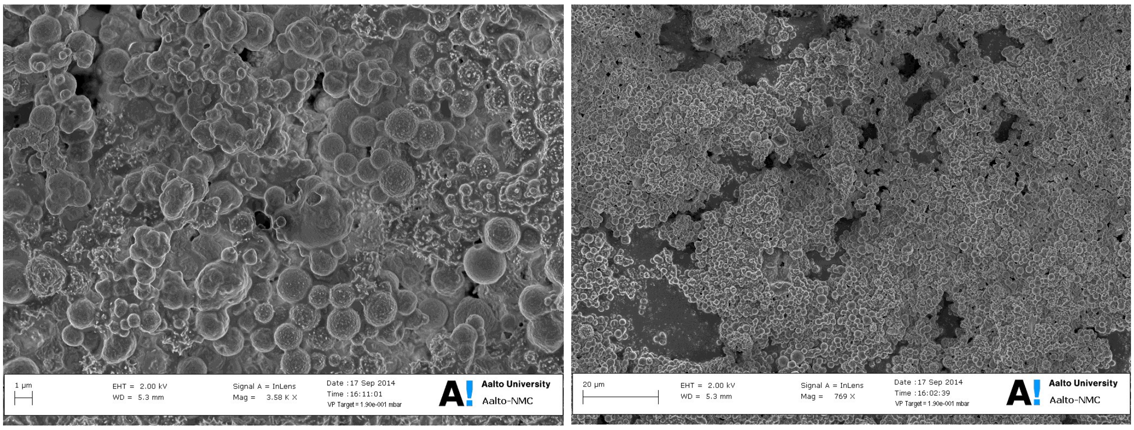

Fig. S25 SEM of CA(AGE$_6$-C$_6$H$_{12}$-UPy)$_4$ in water

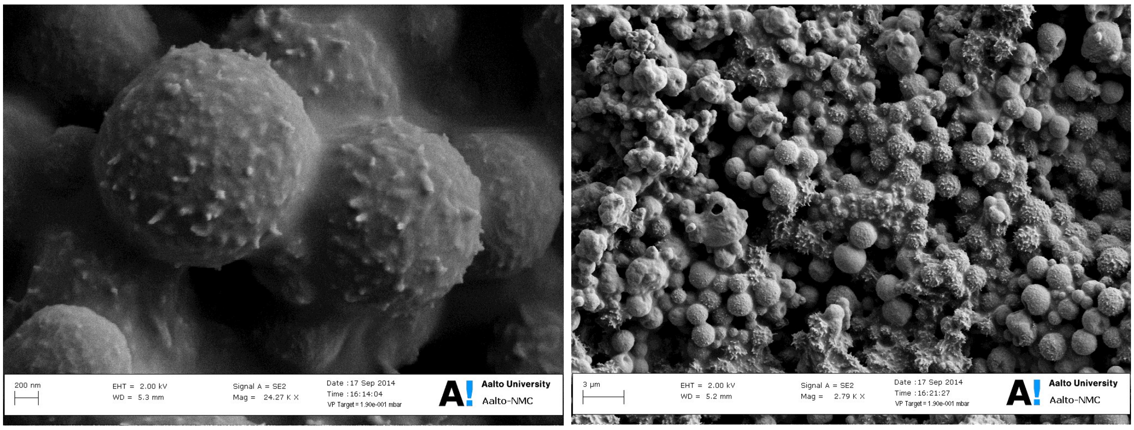

Fig. S26 SEM of CA(AGE$_6$-C$_6$H$_{12}$-UPy)$_4$ in water

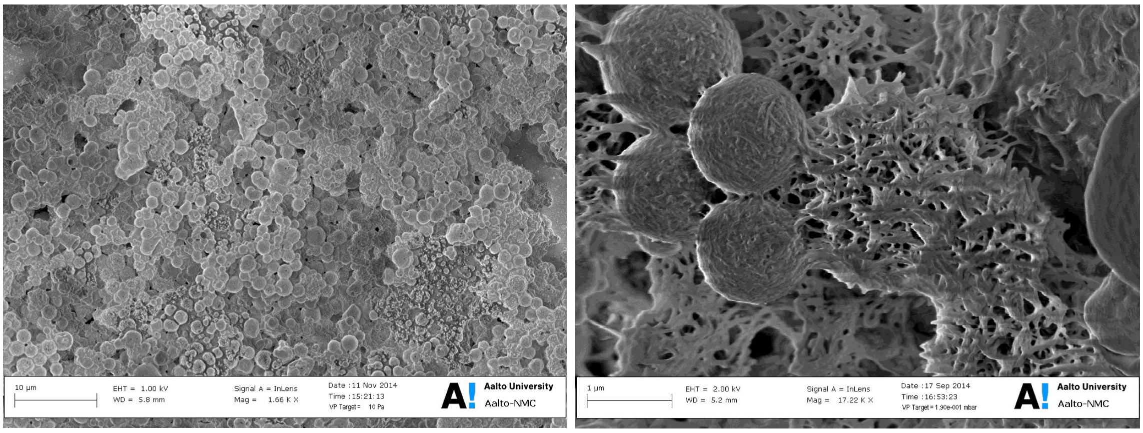

Fig. S27 SEM of CA(AGE$_6$-C$_6$H$_{12}$-UPy)$_4$ in water

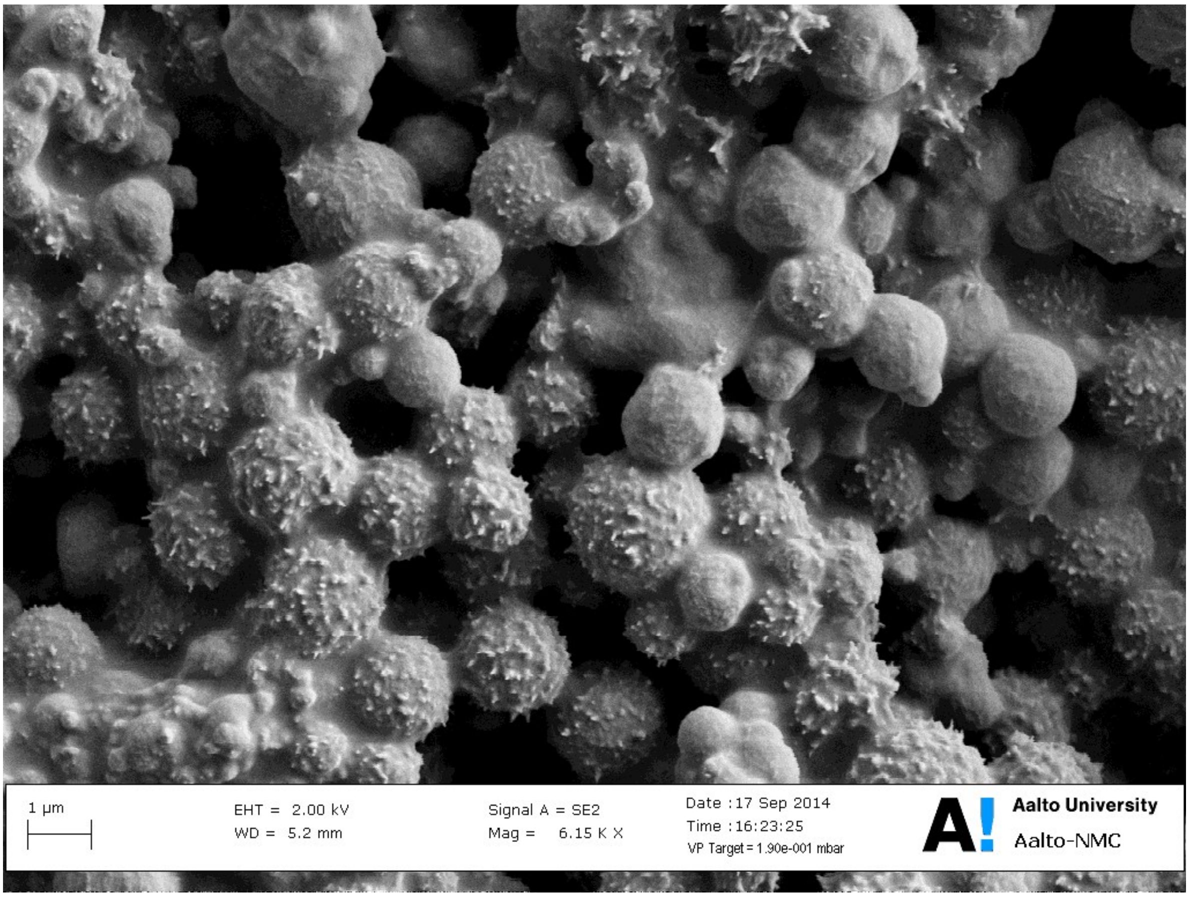

Fig. S28 SEM of CA(AGE_6-C_6H_{12}-UPy)$_4$ in water

9. References:

1 J. Raula, J. Shan, M. Nuopponen, A. Niskanen, H. Jiang, E. I. Kauppinen, H. Tenhu, *Langmuir* 2003**,** ***19***, 3499 – 3504.

2 D. N. Mastronarde, *J. Struct. Bio.* 2005**,** ***152***, 36–51.

3 J. R. Kremer, D. N. Mastronarde, J. R. McIntosh, *J. Struct. Biol*. 1996, ***116***, 71 – 76.

4 G. Giguère, X. X. Zhu, *Biomacromolecules* 2010*,* ***11***, 201-206.
